# Factors influencing breastfeeding practices in China: A meta‐aggregation of qualitative studies

**DOI:** 10.1111/mcn.13251

**Published:** 2021-08-06

**Authors:** Wei Wu, Jian Zhang, Irma Silva Zolezzi, Lisa R. Fries, Ai Zhao

**Affiliations:** ^1^ Vanke School of Public Health Tsinghua University Beijing China; ^2^ Department of Nutrition and Food Hygiene, School of Public Health Peking University Beijing China; ^3^ Nestlé Research Singapore; ^4^ Nestlé Research Beijing China

**Keywords:** breastfeeding, China, influencing factors, policy, qualitative, social network, systematic review

## Abstract

The World Health Organization recommends that women exclusively breastfeed until their babies are 6 months old and continue to breastfeed while introducing complementary foods. A meta‐aggregation methodology was used to systematically review and synthesise the qualitative studies on factors influencing breastfeeding practices of healthy Chinese women in Greater China. English and Chinese databases were searched to identify peer‐reviewed qualitative studies (published 2008–2019). Relevant data were extracted, and key themes related to factors influencing breastfeeding practices were identified. Of 7587 articles identified, 22 qualitative studies met inclusion criteria for the review, 10 of which were published in Chinese. A total of 87 themes were extracted from all included studies and classified into 9 subcategories: government enactment of policies, implementation of policies in workplaces, social expectations, social support, medical and health services, services with Chinese characteristics, breastfeeding and pumping facilities, maternal perceptions of breastfeeding and self‐efficacy to breastfeed. The nine subcategories were then grouped into four categories. Potential effect associations among these influence factors of breastfeeding practices emerged from categories and subcategories. Family members' influence on breastfeeding motivation and self‐efficacy suggest a potential benefit of breastfeeding promotion interventions targeting the whole family. The role of primary care should be fully exploited in breastfeeding promotion, including both prenatal education and post‐partum visits. Standardising the training and qualifications of maternity matrons (*yuesao*) and folk breastfeeding specialists (*cuirushi*) can promote evidence‐based approaches to facilitating breastfeeding during the confinement period. Increased availability of breastfeeding and pumping facilities in the workplace would facilitate continuing breastfeeding after returning to work.

**Key messages**

- The extended family's close relationship to a Chinese mother's breastfeeding self‐efficacy suggests need for education programs further emphasising the nutritional value of breast milk and targeting the whole family.
- There is an opportunity to enhance the positive roles of traditional Chinese maternity matrons and *cuirushi* in promoting breastfeeding: standardising their training on nutrition and care of mothers and children can promote evidence‐based approaches to facilitating breastfeeding.
- Prenatal education and post‐partum visits in community healthcare centres in China could be improved, both in quantity and quality.
- Increased availability of breastfeeding and pumping facilities in the workplace and public places will facilitate women's ability to continue breastfeeding.

## 1 INTRODUCTION

Breastfeeding is known to confer a wide range of health benefits to the infant and the mother, beyond simply providing nutrition. According to the World Health Organization (WHO), breastfeeding was defined as the process of feeding human milk (including milk expressed or from a wet nurse) to an infant, during which any food or liquid including nonhuman milk and formula may be included.. The WHO recommends that infants be exclusively breastfed (where an infant *only* consumes human milk [including expressed milk or from a wet nurse] but may receive oral rehydration salts, drops, or syrups such as vitamins and medicine; WHO, 2008) for the first 6 months of life and continue to be breastfed until two years and beyond (WHO, 2001). Breast milk protects against diarrhoea and respiratory infections and reduces the risk of developing obesity in childhood and adulthood (Victora et al., 2016; Weng et al., 2013). It is noteworthy that breastfeeding can help protect against respiratory infections, especially during the current COVID‐19 pandemic (Lubbe et al., 2020). Protective effects of breastfeeding on breast and ovarian cancer have also been documented (Bartick et al., 2017; Chowdhury et al., 2015).

The exclusive breastfeeding (EBF) rate until 6 months in China has declined from 27.8% in 2008 to 20.8% in 2013 (Center for Health Statistics and Information, 2009; Duan et al., 2018), compared with the average of 37.0% among middle‐ and low‐income countries globally (Victora et al., 2016). A large‐scale study in China found a median duration of breastfeeding was 8.6 months (Yang, Lai, et al., 2016). Another study in 12 regions found that only 65% of 11 months old still received any breast milk (Fang et al., 2020).

Breastfeeding practices are influenced by various intrapersonal and interpersonal factors, as well as by the general social and cultural environment (Andrew & Harvey, 2011; Ayton et al., 2019). Compared with other countries (Cheney et al., 2019; Groleau et al., 2017), traditional post‐partum practices, the family planning policy and the medical care situation have unique effects on breastfeeding practices in China, mediated by family structure and child‐rearing patterns. However, the majority of studies concerning factors influencing breastfeeding in China are quantitative, and they mostly explore direct and superficial factors, such as sociodemographic factors, maternal health or delivery mode (Liu et al., 2013; Waits et al., 2018; Wu et al., 2018). Most of the factors related to breastfeeding identified by quantitative research are unmodifiable, such as maternal age or education. In contrast, qualitative studies tend to identify more specific factors that influence behaviour and are more likely to be modifiable, such as public policy, the home environment, perceptions and facilities.

Although there have been several qualitative studies regarding factors influencing breastfeeding practices in China, some have been published in English and others in Chinese‐language journals. This language gap likely contributed to the absence of a systematic review on this topic. The current review aims to provide a comprehensive overview and an updated insight on factors influencing breastfeeding practices in China, in order to inform the design of locally appropriate breastfeeding promotion strategies.

## 2 METHODS

This qualitative review adopted the meta‐aggregation methodology to synthesise the qualitative studies on factors influencing breastfeeding practices of Chinese healthy adult women in Greater China. Meta‐aggregation aims to examine the essence of texts in a valid manner by maintaining the original meaning and avoiding reviewers' influence on the text. It applies to reviewing qualitative evidence across different studies (Paterson, 2012) and produces descriptive findings with generalizable statements to inform health policymakers and practitioners (Hannes & Lockwood, 2012).

### 2.1 Inclusion and exclusion criteria

The inclusion and exclusion criteria were based on a PICo (population, phenomena of interest and context) strategy. The population was Chinese healthy adult women. The phenomena of interest were factors influencing breastfeeding practices. According to WHO definitions of breastfeeding, we included both exclusive and partial human milk feeding. The context was Greater China, with no other limitations placed regarding cultural background or setting.

Peer‐reviewed original studies published in English or Chinese and focused on breastfeeding (including both exclusive and partial breastfeeding) in a sample of mothers living in Greater China (mainland China, Taiwan, Macao and Hong Kong) and using qualitative methodologies (e.g., focus group, in‐depth interviews) for data collection were included. The exclusion criteria were as follows: studies about Chinese families abroad or families with infants with special needs (e.g., cerebral palsy); studies on adolescent mothers (under 18 years old) or mothers with mental health conditions or communicable diseases (e.g., hepatitis and HIV). Study protocols, social marketing campaigns and overview or commentary papers on maternity care or women's health were also excluded.

### 2.2 Search strategy and study selection

**FIGURE 1:**
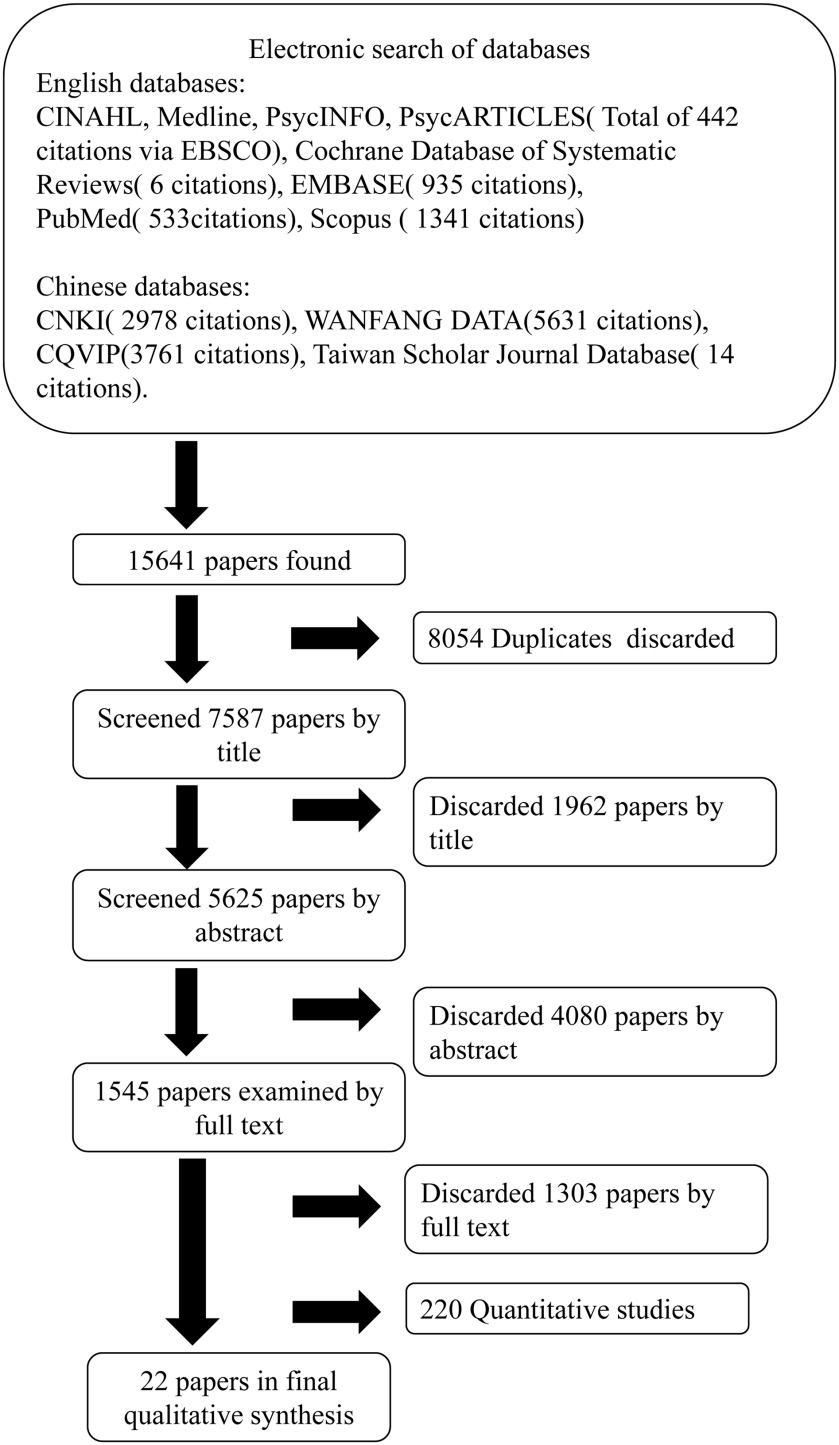
Flowchart showing review process and final number of papers in the report

Two researchers (WW and JZ) conducted an electronic search of eight English databases (CINAHL, Medline, PsycINFO, PsycARTICLES, Cochrane Database of Systematic Reviews, EMBASE, PubMed and Scopus) and four Chinese databases (CNKI, WANFANG DATA, CQVIP and Taiwan Scholar Journal Database). The date range was set between October 2008 and October 2019. Search terms were used to cover the two central themes of the study question: Greater China Region(‘China’, ‘Chinese’, ‘Taiwan’, ‘Taiwanese’, ‘Hong Kong’, ‘Macao’, ‘Macanese’) and breastfeeding(‘breastfeeding’, ‘breast feed’, ‘breast‐feed’, ‘feed’, ‘feeding’, ‘breast feed’, ‘maternal milk’, ‘human milk’, ‘infant feeding’, ‘mother health’, ‘maternal health’ and ‘nursing’). Terms within these two themes were combined with OR, and results from either concept were combined with AND (supporting information Table S1). Reference lists from relevant studies and review papers were also hand searched. Article titles were screened for inclusion or exclusion based on set criteria. Differences in judgement were resolved by discussion between the researchers. The search strategy generated 7587 citations after duplicates were eliminated. This was followed by a screening of all remaining abstracts and then full text versions of papers against the same criteria, after which 1545 full text papers were further examined. After removing 1523 papers, 22 qualitative and mixed methods studies were included in the review. The reference lists of these qualitative studies were also examined, but no further studies fulfilled the inclusion criteria (Figure 1).

### 2.3 Quality assessment

Each qualitative study was assessed using the 2018 Critical Appraisal Skills Programme (CASP) checklist for qualitative research (CASP, 2018). To assess overall study quality, a quality score was calculated by assigning numerical values to each response option (yes = 2, cannot tell = 1, no = 0), and summing across the individual CASP questions, with a maximum possible score of 20 (Dyer et al., 2019).

Critical appraisal of the papers according to the CASP checklist identified several gaps in the quality of the papers overall (supporting information Table S2). Studies were considered ‘good quality’ if the overall CASP score was 16 or higher. One study in the current review had a score of 14, which was the only one with a score below 16. This study was retained in the analysis, as the effect of study quality on certainty of review findings was taken into consideration in the GRADE‐CerQual analyses. Further, consideration of studies with methodological limitations can point to ways of improving future research.

### 2.4 Data extraction and synthesis

Two levels of data extraction were conducted. At the first level, the following data were extracted from each included study: author, year, study location, number of participants, data collection and analysis methods and major factors explored. The second level was about the findings of individual studies, which included the themes reported in individual studies and illustrative quotes from participants, where relevant (supporting information Table S3). In primary studies adopting mixed methods, the findings obtained by qualitative methods were separated from that obtained by quantitative methods, and were extracted in the same way as other qualitative studies.

**FIGURE 2:**
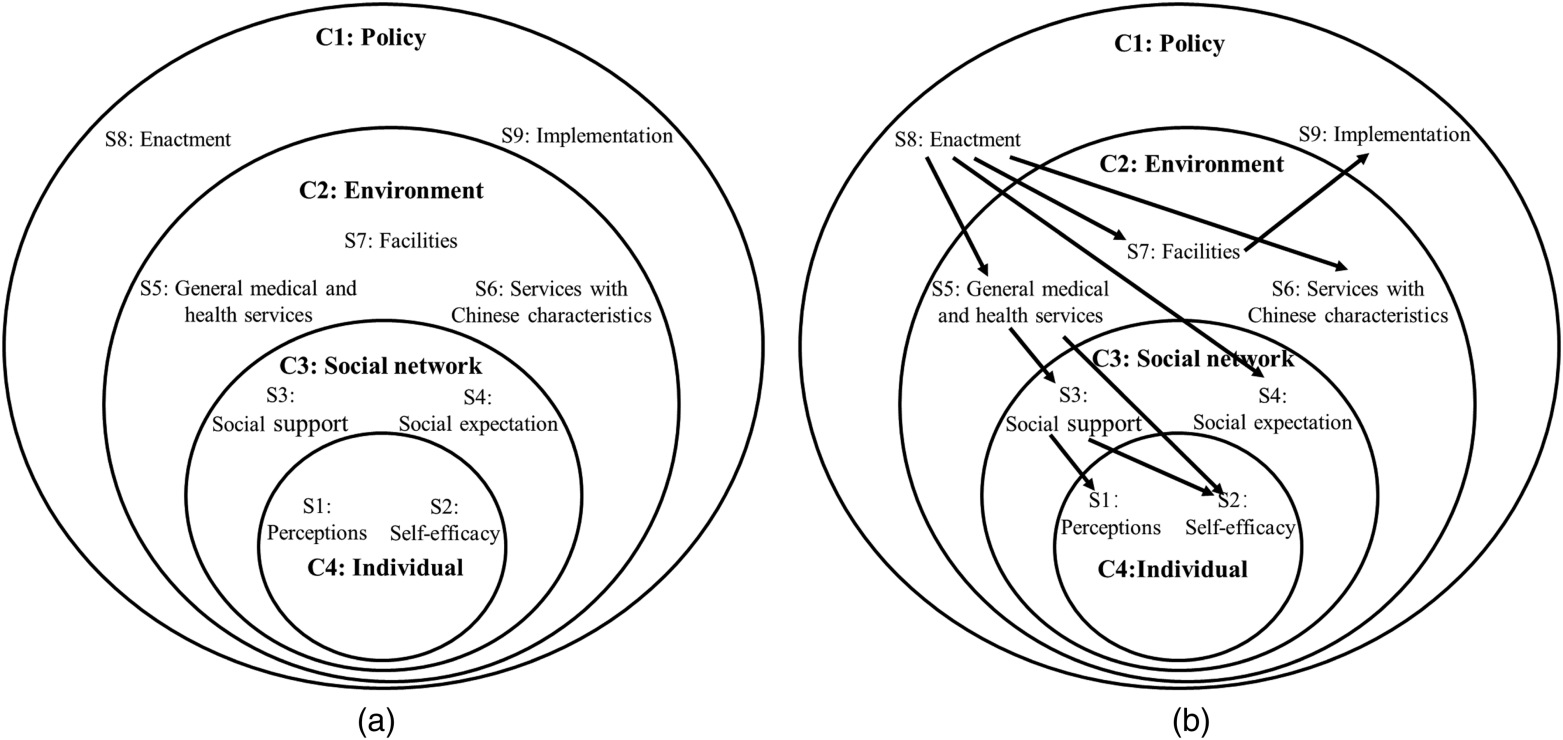
Factors influencing breastfeeding practices. (a) Categories and subcategories of aggregated themes from the included studies. (b) Conceptual framework of association among factors influencing breastfeeding practices. An arrow denoted the hypothesised direction of interaction. C, Category; S, Subcategory

The Joanna Briggs Institute (JBI) method was used to categorise the data, and synthesise the findings (Bath‐Hextall, 2014). The themes, supporting quotes, were aggregated based on the similarity in meaning. Before the data aggregation and synthesis, the first author studied all of the quotations thoroughly. The quotes were arranged into groups and rearranged into subgroups or vice versa until categories became clear. Frequent reference to the articles was performed when necessary to ensure the original meaning of the texts was retained. Based on the different levels of influence on specific health behaviours in the socioecological model (intrapersonal, interpersonal, organisational, community and public policy level) (Quinn et al., 2005), we defined four categories of themes a priori that could relate to factors influencing breastfeeding practices (Bueno‐Gutierrez & Chantry, 2015; Lindsay et al., 2017). The four a priori categories were individual (the intrapersonal level), social network (the interpersonal level), environment (the organisational and community level) and policy (the public policy level). Quotes that could not be classified into the a priori categories were categorised as ‘other’ (Figure 2a). All categories and subcategories were independently checked for accuracy and reviewed by a second researcher to ensure a consensus was reached. Synthesised findings were subsequently formulated from the aggregation and categorisation. All included studies were also categorised according to the subcategories of themes extracted.

### 2.5 Appraisal of certainty of review findings

We used GRADE‐CerQual (certainty of the qualitative evidence) approach to assess the certainty of review findings (Lewin et al., 2018). The overall certainty of each individual review finding was assessed as high, moderate, low or very low, in terms of four components: (1) methodological limitations, (2) coherence, (3) adequacy of data and (4) relevance. The assessments of the four components collectively contribute to an overall assessment of whether findings from a qualitative evidence synthesis provide a reasonable representation of phenomenon of interest.

## 3 RESULTS

### 3.1 Pertinent features of included qualitative studies

A total of 22 papers (19 qualitative studies, 3 mixed methods) were included in this review (Table 1). Of these, 10 were published in Chinese and 12 in English. There were a total of 780 participants across all studies. Of these, 727 were pregnant and/or lactating women, with some studies also including other family members, health care practitioners or policy makers. One study only included fathers. There was a wide range in the number of participants in each study, from 8 mothers in the two smallest studies to 140 in the largest study.

**TABLE 1:** Summary of included qualitative studies

Author, date	Language	Study location		Number of participants			Data collection method	Data analysis method	Study aims	Influence factors
		Name	City tier	Women during pregnancy or having breastfeeding experiences	Others^a^	Total				
Chang et al. (2014)	English	Taiwan		14	0	14	Interviews	Content analysis	To describe the influence of nonfamily‐based support on breastfeeding practices among career women in Taiwan during the first four post‐natal months.	Implementation of policies in workplaces, social expectation, social support, services with Chinese characteristics, facilities
Chen et al. (2019)	English	8 county level regions	Third‐tier	84	0	84	Semistructured interviews	Content analysis	To identify key work‐related factors that influence breastfeeding practices in Chinese working mothers.	Government's enactment of policies, implementation of policies in workplaces, social expectation, social support, facilities, self‐efficacy
Chen et al. (2016)	Chinese	Ningbo, Zhejiang	Third‐tier	0	12	12	In‐depth interviews	Colaizzi's phenomenological analysis	To understand paternal views of breastfeeding and paternal experiences of supporting and being involved in breastfeeding. To let medical workers offer fathers effective and timely assistance, make fathers play a full role in breastfeeding and facilitate practice of breastfeeding.	Social support, general medical and health services, perceptions, self‐efficacy
Chen et al. (2016)	English	Chengdu, Sichuan	First‐tier, rural	21	0	21	Focus group discussions	Thematic analysis	To assess infant feeding practices in urban and rural areas of the Deyang region.	Social support, general medical and health services, perceptions, self‐efficacy
Hanser and Li (2017)	English	Shanghai	First‐tier	51	0	51	Interviews	Issue‐centred analysis	To explore the relationship between cultural definitions of good mothering and breastfeeding among middle‐class, urban Chinese women.	Government's enactment of policies, perceptions
Ho and McGrath (2011)	English	Taiwan		140	0	140	Interviews	Content analysis	Mothers' reasons for changing their infant feeding method from breastfeeding to bottle feeding and their perceived social support.	Social support
Hu et al. (2013)	Chinese	Shanghai	First‐tier	8	0	8	Semistructured interviews	Not mentioned	To understand the status of mothers' return to work and supportive status of breastfeeding in workplace in Shanghai.	Government's enactment of policies, facilities
Jiang et al. (2012)	English	Shanghai	First‐tier	38	0	38	In‐depth interviews and focus group discussions	Content analysis	To explore mothers' awareness of the WHO guidelines for breastfeeding and their intention to breastfeed. To identify the gap between mothers' needs and perinatal care provision for breastfeeding.	General medical and health services, facilities, perceptions
Li et al. (2014)	Chinese	Wuhan, Hubei	Second‐tier	16	0	16	In‐depth interviews	Colaizzi's phenomenological analysis	To understand the breastfeeding experience and needs of professional women within one year post‐partum and to generate corresponding countermeasures.	Government's enactment of policies, implementation of policies in workplaces, social expectation, social support, general medical and health services, facilities, perceptions, self‐efficacy
Ouyang et al. (2016)	English	Wuhan, Hubei	Second‐tier	76	0	76	In‐depth interviews	Not mentioned	To explore the difficulties and desires of Chinese breast‐feeding women.	General medical and health services, services with Chinese characteristics, perceptions
Tarrant et al. (2014)	English	Hong Kong		24	0	24	In‐depth interviews	Content analysis	To explore the breast‐feeding experiences of Hong Kong Chinese mothers who prematurely discontinue breastfeeding and to identify contributing factors that might be remediated to help women breast feed longer.	Government's enactment of policies, social support, general medical and health services, perceptions, self‐efficacy
Wu et al. (2008)	English	Taiwan		10	0	10	Interviews	Content analysis	To describe the experiences of ten three‐shift nurses, with particular focus on how they make arrangements regarding breastfeeding in relation to their workplaces and work breaks.	Implementation of policies in workplaces, social expectation, facilities
Wu et al. (2017)	Chinese	Shenzhen, Guangdong	Second‐tier	28	0	28	In‐depth interviews	Colaizzi's phenomenological analysis	To explore experiences of outpatient visits and the need for breastfeeding support of pluriparas who failed to breastfeed.	General medical and health services, services with Chinese characteristics, perceptions, self‐efficacy
Yan et al. (2018)	Chinese	Unknown		15	0	15	Semistructured interviews	Colaizzi's phenomenological analysis	To explore experiences of professional women breastfeeding in the work environment.	Implementation of policies in workplaces, social expectation, facilities, perceptions
Yang et al. (2011)	Chinese	Shanghai	First‐tier	23	0	23	Semistructured interviews	Not mentioned	To understand breastfeeding knowledge of pregnant women and young mothers, and their attitude about being involved in a breastfeeding promotion program by text messages and the internet.	General medical and health services
Yang et al. (2015)	Chinese	Yichang, Hubei	Third‐tier	24	0	24	In‐depth interviews	Not mentioned	To provide the basis for medical workers to offer breastfeeding guidance, help mothers of twins cope with difficulties and boost the breastfeeding rate.	General medical and health services, perceptions, self‐efficacy
Yang, Liu, et al. (2016)	Chinese	Yichang, Hubei	Third‐tier	19	0	19	In‐depth interviews	Not mentioned	To investigate the need for help and support from the partner in the perspective of mothers.	Social support
Yu et al. (2018)	Chinese	Qingdao, Shandong	Second‐tier	11	0	11	In‐depth interviews	Colaizzi's phenomenological analysis	To explore experiences of successful breastfeeding and needs of primiparas who had a natural delivery.	Social support, general medical and health services, services with Chinese characteristics, perceptions, self‐efficacy
Yu et al. (2013)	Chinese	Shanghai	First‐tier	8	0	8	In‐depth interviews	Colaizzi's phenomenological analysis	To explore how primiparas feel about attending the breastfeeding counselling clinic under the guidance of midwives or nurses with a family member.	General medical and health services
Zhang et al. (2015)	English	Hangzhou, Zhejiang and Shenzhen, Guangdong	Second‐tier	50	33	83	In‐depth interviews and focused group discussions	Thematic analysis	To elicit and compare mothers' and hospital staff perceptions of the reasons that shaped mothers' decision to formula feed.	Government's enactment of policies, implementation of policies in workplaces, social expectation, social support, general medical and health services, services with Chinese characteristics, facilities, perceptions, self‐efficacy
Zhang et al. (2018)	English	Shanghai and Weifang, Shandong	First‐tier and third‐tier	40	8	48	In‐depth interviews and focused group discussions	Content analysis	To elicit Chinese mothers' current breastfeeding experiences; to explore if the current breastfeeding service delivery meets mothers' demands, and their needs for breastfeeding support in two cities‐Shanghai and Weifang, China.	Implementation of policies in workplaces, social expectation, social support, general medical and health services, services with Chinese characteristics, facilities, perceptions, self‐efficacy
Zhao et al. (2018)	English	Wuhan, Hubei	Second‐tier	27	0	27	Interviews	Content analysis	To explore the experiences and expectations of breast‐feeding mothers in public places in Wuhan, China.	Government's enactment of policies, facilities

The majority of qualitative studies (13/22) come from first‐tier and second‐tier cities, along with five studies covering a third‐tier city and one study from a rural region. Four of the studies were from Hong Kong or Taiwan. City tiers are defined based on a variety of factors, with tier 1 representing the largest and wealthiest cities with the strongest infrastructure and commercial opportunities (Xu, 2019).

The most commonly used data collection methods were in‐depth interviews (IDI; used as sole method in nine studies) and (semistructured) interviews (nine studies). Three studies combined IDI and focus group discussions (FGD), and one study used only FGD.

The data analysis methods for the studies varied, with eight studies using content analysis, six using [Colaizzi's] phenomenological analysis, two using thematic analysis and one using issue‐centred analysis. The remaining five papers did not state the theoretical framework adopted.

### 3.2 Description of findings

A total of 87 themes were extracted, which were then clustered into 9 subcategories, and then grouped into 4 categories: individual factors, social network, environment and policy. Potential associations among these factors emerged between categories and subcategories, as illustrated in Figure 2b.

The two most commonly mentioned subcategories (13 studies) were perceptions of breastfeeding and general medical and health services. Services with Chinese characteristics, such as home help during the confinement period, were the least often discussed (six studies). (supporting information Figure S1).

The CERQual assessment demonstrated high confidence in the evidence for the following subcategory findings: perceptions, self‐efficacy, social support and general medical and health services (Table 2 and supporting information Table S4). There is moderate confidence in the evidence for social expectations, facilities, government enactment of policies and implementation of policies in the workplace. Confidence was low for findings related to services with Chinese characteristics.

**TABLE 2:** CERQual summary of qualitative findings

Summary of the review finding	Studies contributing to the review finding	CERQual assessment of confidence in the evidence	Explanation of CERQual assessment
1. Perceptions: Mothers commonly perceive that breast milk is equally or even less nutritious than formula, or breast milk supply is insufficient. Many women recognised that breastfeeding is beneficial for infant and maternal health. Some women found breastfeeding more difficult than expected.	Chen et al. (2016); Gao et al. (2016); Hanser and Li (2017); Jiang et al. (2012); Li et al. (2014); Ouyang et al. (2016); Tarrant et al. (2014); Wu et al. (2017); Yan et al. (2018); Yang, Lai, et al. (2016); Yu et al. (2018); Zhang et al. (2015); Zhang et al. (2018)	High confidence	Moderate methodological limitations and minor concerns regarding relevance. No or very minor concerns about coherence and adequacy.
2. Self‐efficacy: Women's breastfeeding practice was influenced by their own and others' experiences with breastfeeding, physical factors, challenges encountered when returning to work.	Chen et al. (2019); Chen et al. (2016); Gao et al. (2016); Li et al. (2014); Tarrant et al. (2014); Wu et al. (2017); Yang et al. (2015); Yu et al. (2018); Zhang et al. (2015); Zhang et al. (2018)	High confidence	Moderate methodological limitations and minor concerns regarding relevance. No or very minor concerns about coherence and adequacy.
3. Social expectation: Most mothers reported coworkers' positive attitudes towards breastfeeding. In some cases, employers were not supportive. Some mothers mentioned unfriendly atmosphere about breastfeeding in public.	Chang et al. (2014); Chen et al. (2019); Li et al. (2014); Wu et al. (2008); Yan et al. (2018); Zhang et al. (2015); Zhang et al. (2018)	Moderate confidence	Moderate methodological limitations and minor concerns regarding relevance and adequacy. No or very minor concerns about coherence.
4. Social support: Almost all mothers described that they had received physical or psychological support to breastfeeding from their spouse. Both encouragement and discouragement of breastfeeding from mother and mother‐in‐law were common, depending on their own experiences and perceptions of feeding.	Chang et al. (2014); Chen et al. (2019); Chen et al. (2016); Gao et al. (2016); Ho and McGrath (2011); Li et al. (2014); Tarrant et al. (2014); Yang, Lai, et al. (2016); Yu et al. (2018); Zhang et al. (2015); Zhang et al. (2018)	High confidence	Moderate methodological limitations and minor concerns regarding relevance. No or very minor concerns about coherence and adequacy.
5. General medical and health services: One nurse and many mothers reported limited guidance on breastfeeding skills in the hospital after delivery. Many participants reported the positive role of outpatient breastfeeding consultations on breastfeeding confidence. In contrast, some women reported that medical staff in the community level lacked professional guidance on breastfeeding.	Chen et al. (2016); Gao et al. (2016); Jiang et al. (2012); Li et al. (2014); Ouyang et al. (2016); Tarrant et al. (2014); Wu et al. (2017); Yang et al. (2011); Yang et al. (2015); Yu et al. (2018); Yu et al. (2013); Zhang et al. (2015); Zhang et al. (2018)	High confidence	Moderate methodological limitations and minor concerns regarding relevance. No or very minor concerns about coherence and adequacy.
6. Services with Chinese characteristics: Maternity matron and *cuirushi* were considered helpful to breastfeeding, while in some cases they were reported to let mothers feed the baby infant formula or delivered misinformation about breastfeeding.	Wu et al. (2017); Yu et al. (2018); Zhang et al. (2018); Chang et al. (2014); Zhang et al. (2015); Ouyang et al. (2016);	Low confidence	Moderate methodological limitations, and moderate concerns regarding adequacy and relevance. No or very minor concerns about coherence.
7. Facilities: The lack of designated public places for breastfeeding, or places for expressing and storing breast milk in workplaces was listed as a barrier to breastfeeding.	Chang et al. (2014); Chen et al. (2019); Hu et al. (2013); Jiang et al. (2012); Li et al. (2014); Wu et al. (2008); Yan et al. (2018); Zhang et al. (2015); Zhang et al. (2018); Zhao et al. (2018)	Moderate confidence	Moderate methodological limitations and minor concerns regarding relevance. No or very minor concerns about coherence and adequacy.
8. Government enactment of policies: Although mothers referred to maternity leave and breastfeeding leave policies as promoting breastfeeding, many mentioned that a one‐hour breastfeeding break was not long enough. Some mentions of the one‐child policy and the increased involvement of the extended family in feeding a single child may act as a barrier to breastfeeding.	Zhao et al. (2018); Tarrant et al. (2014);Hanser and Li (2017); Hu et al. (2013); Li et al. (2014); Zhang et al. (2015); Chen et al. (2019)	Moderate confidence	Moderate methodological limitations, moderate concerns regarding relevance, and minor concerns about adequacy and coherence.
9. Implementation of policies in the workplace: Many mothers reported breastfeeding breaks could not be guaranteed mainly due to difficulties in finding places and time to breastfeed during busy work schedules.	Chang et al. (2014); Chen et al. (2019); Li et al. (2014); Wu et al. (2008); Yan et al. (2018); Zhang et al. (2015); Zhang et al. (2018)	Moderate confidence	Moderate methodological limitations, minor concerns regarding relevance. No or very minor concerns about adequacy and coherence.

#### 3.2.1 Individual

##### Perceptions

By synthesising findings across studies, we identified three main types of maternal perceptions about breastfeeding: comparisons between breast milk and infant formula (IF), impact of breastfeeding on infant and maternal health and expectations about breastfeeding.

Maternal perceptions about the relative advantages and disadvantages of breast milk and IF related to both quality and quantity. The perception that breast milk is equally or even less nutritious than formula was common across studies. Mothers holding this belief generally perceived that (1) breast milk becomes less nutritious as time passes, which three mothers reported learning from older female relatives; (2) nutrients in formula are ‘clearly labeled on the packaging’ (Zhang et al., 2015) while breast milk components are invisible and unfamiliar; some mothers cited needing to supplement breast milk (e.g., with vitamin D drops) in their concerns about whether breast milk provided sufficient nutrition for the baby; and (3) growth and development of formula‐fed infants is not inferior to breastfed ones. Another common perception was that of insufficient breast milk supply, because mothers found it difficult to judge if a breastfed child was full. It was easy to quantify formula consumption, while a lot of breastfeeding mothers were unable to tell how much the child had consumed or tended to link crying to hunger.

Although many women recognised that breastfeeding is beneficial for infant and maternal health, with a focus on parent–child bonding, only a minority could specify other benefits, such as supporting baby's immune system or reducing the mother's cancer risk (Jiang et al., 2012; Yan et al., 2018). Moreover, some women believed in a relationship between breast milk and jaundice or that breastfeeding was unfavourable to losing pregnancy weight, which negatively affected breastfeeding practices (Gao et al., 2016; Hanser & Li, 2017; Yu et al., 2018).

There were gaps between expectations of breastfeeding and reality. Some women expected breastfeeding to be ‘natural’ (Tarrant et al., 2014; Zhang et al., 2018) and were unprepared for potential difficulties.

##### Self‐efficacy

Breastfeeding self‐efficacy is defined as a mother's confidence in her ability to breastfeed her infant (Dennis, 2003). Women's breastfeeding self‐efficacy was influenced by both their own experiences with breastfeeding, and those of relatives and friends. Physical factors, such as pain due to caesarean section, could cause negative breastfeeding experiences and were a barrier to breastfeeding, especially in the first days post‐partum.

Returning to work was a transitional time when many challenges emerged that negatively impacted breastfeeding self‐efficacy. Numerous studies cited exhaustion due to juggling breastfeeding and work and sleep‐deprivation among the main reasons for supplementing with formula or completely stopping breast feeding.

#### 3.2.2 Social network

##### Social expectation

The expectations of employers and co‐workers about breastfeeding influenced breastfeeding practices, especially when women went back to work. Positive attitudes of co‐workers were observed in the majority of studies, in the form of verbal encouragement and offers to help with professional tasks. However, in three studies which mentioned managers (Li et al., 2014; Zhang et al., 2015, 2018), supportive attitudes were less evident.

Two studies mentioned expectations about breastfeeding in public. Among the mothers in these studies, only one called for ‘eliminating discrimination’, while others said they avoided breastfeeding in public for fear of being considered ‘morally deficient’, or ‘indecent’ (Zhang et al., 2018; Zhao et al., 2018).

##### Social support

Of mothers who discussed the role of the spouse, almost all reported that their spouse was supportive of breastfeeding, which could be demonstrated in different ways. In cases where both parents received breastfeeding education together, the father could give advice when challenges arose during breastfeeding. Husbands that understood the importance of breastfeeding were also more willing to lighten the mother's workload by sharing childcare and household duties. Two studies also mentioned fathers being attentive to their spouse's mood and providing psychological support.

Breastfeeding support from elders principally came from the mother and mother‐in‐law (MMIL). Although some mothers mentioned that MMIL were supportive of breastfeeding, our synthesis found that it was still common for the MMIL to discourage breastfeeding, mostly based on MMIL's own experiences and perceptions of feeding. MMIL often believed that feeding IF made children grow faster, especially in terms of weight gain, and made it easier to quantify how much milk had been fed.

#### 3.2.3 Environment

##### General medical and health services

Although a few mothers mentioned ‘prenatal classes’ (Jiang et al., 2012; Yang et al., 2015), only two reported that they ‘learned about the benefits of breastfeeding’ or ‘knew EBF should last for 6 months’ from prenatal education (Jiang et al., 2012).

Many quotes reflected a lack of effective or immediate guidance on breastfeeding skills when women were in the hospital after delivery, which was attributed to short hospital stays and busy medical staff. Mothers' experiences and satisfaction with breastfeeding support from post‐partum medical care depended on the type of practitioner they saw. Two studies highlighted the positive role of outpatient breastfeeding consultations, with many participants reporting that they provided useful knowledge and solutions for breastfeeding difficulties. Some women reported that these consultations improved their breastfeeding confidence through learning, for example, ‘how to judge whether babies are full’, ‘how to continue breastfeeding after returning to work’ and ‘massages and physical therapies’(Li et al., 2014; Wu et al., 2017). In contrast, some women reported that their ‘general practitioners’ or ‘paediatricians’ in the community level sometimes lacked ‘professional’ guidance on breastfeeding (Chen et al., 2016; Zhang et al., 2018). Most community doctors simply inquired about ‘the way of feeding’ without providing feedback or suggested to ‘supplement with formula after concluding that the baby was underweight’ (Chen et al., 2016; Jiang et al., 2012; Yang et al., 2011; Zhang et al., 2018). Furthermore, two studies found that mothers desired more post‐partum visits.

##### Services with Chinese characteristics

According to Chinese tradition, new mothers stay home and rest for a ‘confinement period’ of about 1 month (28–42 days) after giving birth, which is thought to facilitate recovery. During this period, a maternity matron (*yuesao*) may be hired to prepare traditional post‐partum foods and help with household and childcare tasks. New mothers who experience problems with breast milk volume or breast pain may also hire a *cuirushi* to do traditional massage or acupressure, and to prepare galactagogues, such as special soups based on traditional Chinese medicine, to promote milk production. Maternity matron and *cuirushi* were considered helpful to breastfeeding as they promoted lactation through breast massage, encouraging frequent suckling or other Chinese traditional methods. However, some mothers still mentioned that the maternity matron and *cuirushi* let them feed the baby IF, because they ‘do not really understand breastfeeding’ or ‘felt it was more convenient to feed with formula’ (Zhang et al., 2018). Another study displayed the concern that new mothers might receive misinformation from the maternity matron or *cuirushi* (Wu et al., 2017).

A modern alternative to spending the confinement period at home is to stay at a dedicated ‘confinement centre’ for new mothers (*yuezizhongxin*) with round the clock care and pampering. Only one study addressed *yuezizhongxin*, in which two mothers stated that they chose to stay in at the confinement centre because they thought they would receive more breastfeeding support there than from family.

##### Facilities

The lack of designated public places where mothers could breastfeed resulted in unpleasant breastfeeding experiences, with mothers feeling ‘worried’ or ‘uncomfortable’ (Zhang et al., 2015; Zhao et al., 2018) when breastfeeding in public. Workplaces were also described as not being breastfeeding friendly by most working mothers, because it was difficult to find appropriate places for expressing breast milk or available refrigerators for storing the milk. Some lactating mothers used a restroom or locker room to continue breastfeeding after resuming work, while others had to switch to mixed feeding or discontinue breastfeeding.

#### 3.2.4 Policy

##### Government's enactment of policies

Maternity leave covering a period of at least 14 weeks is regulated at the national level, of which 2 weeks can be taken before the birth. Some provinces have local allowances for additional leave of about 2–6 weeks for a total maternity leave of 4–5 months. After returning to work, through the baby's first birthday, mothers are entitled to two half‐hour breaks each day to breastfeed or express breast milk. Across studies, mothers referred to these maternity leave and breastfeeding leave policies as promoting breastfeeding. However, many mothers discussed challenges related to the practical aspects of these policies. Most women mentioned that a 1‐h breastfeeding break was not long enough for them to breastfeed babies directly (e.g., due to living far from the workplace), so that some chose to express breast milk instead.

In addition, two studies suggested that the one‐child policy may have had an indirect impact on breastfeeding. Starting in 1980, families in China were limited to having a single child, and only in the past few years has it become possible to have a second baby, although it is still relatively uncommon for families to take advantage of this new option. As a result, most babies are both ‘only children’ and ‘only grandchildren’. An obstetrics nurse and a mother both mentioned that the one‐child policy and the increased involvement of the extended family in feeding a single child may act as a barrier to breastfeeding.

##### Implementation in the workplace

Only one study reported satisfaction with the implementation of breastfeeding policies in workplaces, giving examples such as women not being asked to take business trips during the lactation period (Yan et al., 2018). Other studies found that breastfeeding breaks could not be guaranteed, even if mothers intended to express breast milk during this break. Difficulties in finding places to breastfeed and time during busy work schedules were listed as the main reasons. Some mothers reported stopping breastfeeding earlier than planned due to heavy workloads. Two studies focusing on nurses highlighted the unfavourable influence of graveyard shifts on breastfeeding.

## 4 DISCUSSION

To the best of our knowledge, this is the first systematic review of qualitative studies on factors influencing breastfeeding practices of mothers living in Greater China, including both Chinese and English language articles. Themes extracted from the included studies were grouped into four categories: individual factors, social network, environment and policy. Our review identified maternal perceptions and self‐efficacy, facilities in public places (including work places), support provided during medical health services, social expectations and policy as factors influencing breastfeeding in China, similar to findings of reviews from other countries (e.g., Carpenter et al., 2016; De Roza et al., 2019; Galipeau et al., 2018; Glassman et al., 2014; Wainaina et al., 2018). However, family members' high level of involvement in breastfeeding practices in a one‐child family structure, as well as the role of the maternity matron and *cuirushi* may be specific to Chinese culture and society. Potential associations among these factors emerged from these findings, as illustrated in Figure 2b. Returning to work was a critical transition period and often a cause of early cessation of EBF (Barbosa et al., 2017; Kuswara et al., 2016). Breastfeeding self‐efficacy can be undermined by stress and fatigue from juggling work and breastfeeding, especially when the work environment is not supportive. Simple measures such as lactation rooms and nursing breaks are low‐cost interventions that could promote breastfeeding (Addati et al., 2014). The Chinese government has already enacted several positive policies to promote breastfeeding for working women, including paid maternity leave and breastfeeding breaks. However, there is still room for improvement in the execution of these policies to make them more effective, such as by requiring employers to provide facilities for breastfeeding or pumping milk. There is also some debate about the length and flexibility of breastfeeding breaks, as mothers reported that 1 h was not long enough to breastfeed in person. Chinese mothers are currently entitled to a maternity leave from 4 to 5 months after delivery (Xiao & Lin, 2018). Given the recommendation to breastfeed exclusively for 6 months and to continue breastfeeding until 2 years and beyond, a strong workplace policy to support continued breastfeeding would help mothers to reach this goal, even after returning to work. Policies that promote breastfeeding may also influence social acceptability and expectations about breastfeeding (Bueno‐Gutierrez & Chantry, 2015).

Support from healthcare practitioners is another important contributor to breastfeeding self‐efficacy in our review, similar to findings from a study in New Zealand (Alianmoghaddam et al., 2017). Support provided in both hospital and community settings from the antenatal to post‐natal period has been shown to increase EBF rates up to 6 months elsewhere (Kang et al., 2018; Kim et al., 2018). However, in our review, prenatal breastfeeding promotion from health professionals was only mentioned by a handful of mothers. As gaps between expectations of breastfeeding and difficulties encountered were identified in our review, mothers might benefit from more prenatal advice to prepare them for potential breastfeeding challenges. The access to breastfeeding guidance was limited during the post‐delivery hospital stay, which may be a result of the relatively low doctor: patient ratios (Song et al., 2020). For this reason, post‐partum home visits and outpatient consultations are key sources of breastfeeding guidance, especially for new mothers to ask questions and resolve problems in a timely manner. The traditional Chinese ‘confinement period’ also amplifies the significance of post‐partum home visits for promoting breastfeeding. Unfortunately, our review found that post‐partum visits in the primary care setting were sometimes unsatisfactory, with some mothers reporting insufficient guidance on breastfeeding. Post‐partum visits could be an important opportunity to strengthen social support to breastfeeding by involving both the mother and other members of her family. Only the post‐natal visit on day 42 is regulated currently in China (Xiao & Lin, 2018). Therefore, increasing the frequency and quality of post‐natal visits, especially in the early post‐partum period, could be beneficial.

Our results expanded upon previous themes with new findings regarding services with Chinese characteristics, which were linked to traditional post‐natal rituals, such as the ‘confinement period’. A few mothers in our review implied that the maternity matron or *cuirushi* may have had a negative effect on breastfeeding. With little formal professional training, these service‐providers may pass on misconceptions about infant feeding to mothers or encourage mothers to use IF. There is currently no standardised training or qualification system for maternity matrons and *cuirishi*, but drawing lessons from the international board certified lactation consultants (IBCLC) (‘Current Statistics on Worldwide IBCLCs’, 2020), standardising this training and certification process could promote evidence‐based approaches to facilitating breastfeeding during the confinement period (Chen & Feng, 2020). There is an opportunity for Chinese maternity matrons and folk breastfeeding specialists to become a valuable resource by providing breastfeeding counselling.

Social support is also a key factor influencing breastfeeding self‐efficacy (Ngo et al., 2019; Vinderola et al., 2007). In our review, the role of family support was found to be amplified by Chinese child‐rearing patterns. In China, a two‐child policy is progressively being put in place, but there are lasting implications of one‐child policy on Chinese family structure and child‐rearing patterns. The one‐child family structure can intensify attention from the extended family, especially the spouse and grandmothers, who are the people that most often influence maternal decisions and self‐efficacy to breastfeed. Our study revealed that the grandmothers often discouraged breastfeeding, mainly based on their own experiences and perceptions. These findings are consistent with a review of the literature across 35 different countries, finding that senior women or grandmothers often advised new parents and imparted their breastfeeding knowledge to the family (Aubel, 2012). Accordingly, it is important to recognise the roles of senior women in promoting breastfeeding when considering intervention targets; however, educational interventions currently typically target the pregnant or lactating mother without including her social network (Oliveira et al., 2017). For the role of spouse, our review further found that not only verbal encouragement but also physical assistance with other childcare and household duties enhanced breastfeeding self‐efficacy (Dennis, 2003; Schindler‐Ruwisch et al., 2019).

The social network also plays a key role in forming mothers' perception about breastfeeding. Our review found that many mothers hold misconceptions or concerns about the nutritional value of breast milk. Many of them are traditional beliefs passed down by older female relatives. To address some of these maternal concerns related to breast milk, future educational initiatives should underline the nutritional aspects of breastfeeding. This could include information about the different components of breast milk, how breast milk composition changes over time and recommendations for maternal diet. It could also highlight the health benefits of breast milk for infant development. Including the spouse and other relatives might improve the impact of breastfeeding education interventions (Abbass‐Dick et al., 2019).

The current qualitative review was performed according to accepted guidelines, including a critical appraisal of individual study reporting quality and using appropriate synthesis methods. However, this systematic review is not without its limitations. Firstly, the heterogeneity in the quality of the included studies somewhat limits the generalizability of results. This demonstrates the need for more quality research, including pilot tested interview guides and considering feedback on findings from participants. Secondly, most studies included are conducted in southern areas and relatively developed areas of China. Considering the effect of social and cultural context on breastfeeding practices within China, studies covering broader geographical regions and rural areas would provide additional insights. Thirdly, there was heterogeneity in the research objectives/questions and the methodology used across studies, which could have influenced the factors identified. For example, some studies specifically recruited working women and may therefore have elicited more themes related to the workplace than research that included stay‐at‐home mothers. Further, the studies were not consistent in whether they focused on exclusive and/or partial breastfeeding, and for some publications, a formal definition of breastfeeding was not provided. Although there are likely different factors associated with different human milk feeding behaviours (e.g., initiation, exclusivity and duration), these could not be disentangled in the current review. Thematic analysis is a relatively simple approach, which does not focus on the similarities and differences between the studies nor on the theoretical aspects of the findings. A formal compatibility assessment was not conducted. Future research which addresses these gaps could deepen our understanding.

## 5 CONCLUSIONS

This review explored the factors influencing breastfeeding behaviours by meta‐aggregation of qualitative studies and used a conceptual framework to illustrate potential pathways via which these factors interact with each other. Among these factors, the extended family's involvement in breastfeeding practices in a one‐child family structure, as well as the Chinese traditional approaches used by Chinese maternity matrons and *cuirushi*, may be specific to China, compared with other countries. Education programs emphasising the nutritional value of breast milk and targeting the important role of the whole family are needed in achieving breast feeding success. There is an opportunity to enhance the positive roles of traditional Chinese maternity matrons and *cuirushi* in promoting evidence‐based approaches to facilitating breastfeeding, by standardising their training and certification. Prenatal education and post‐partum visits in community healthcare centres in China could be improved, both in quantity and quality. Increased availability of breastfeeding and pumping facilities in the workplace and public places will facilitate women's ability to continue breastfeeding.

## CONFLICTS OF INTEREST

ISZ and LRF are employees of Societé des Produits Nestlé S.A. (SPN). None of the other authors report any potential conflict of interest.

## CONTRIBUTIONS

WW, JZ and AZ performed the research and wrote the paper. ISZ and LRF designed the research and revised the paper.

## Supporting information

**Figure S1.** Supporting Information

**Table S1** Search strategy

**Table S2** Summary of CASP assessment of the included papers ^a^

**Table S3a** Full thematic analysis

**Table S4** CERQual Qualitative Evidence Profile

## Data Availability

Data sharing is not applicable to this article as no datasets were generated or analysed during the current study.
