# Supplementary figures and images for: Factors influencing breastfeeding practices in China: A meta‐aggregation of qualitative studies
Source: Matern Child Nutr. 2021 Aug 6;17(4):e13251. doi: 10.1111/mcn.13251 (PMC8476444; doi:10.1111/mcn.13251)

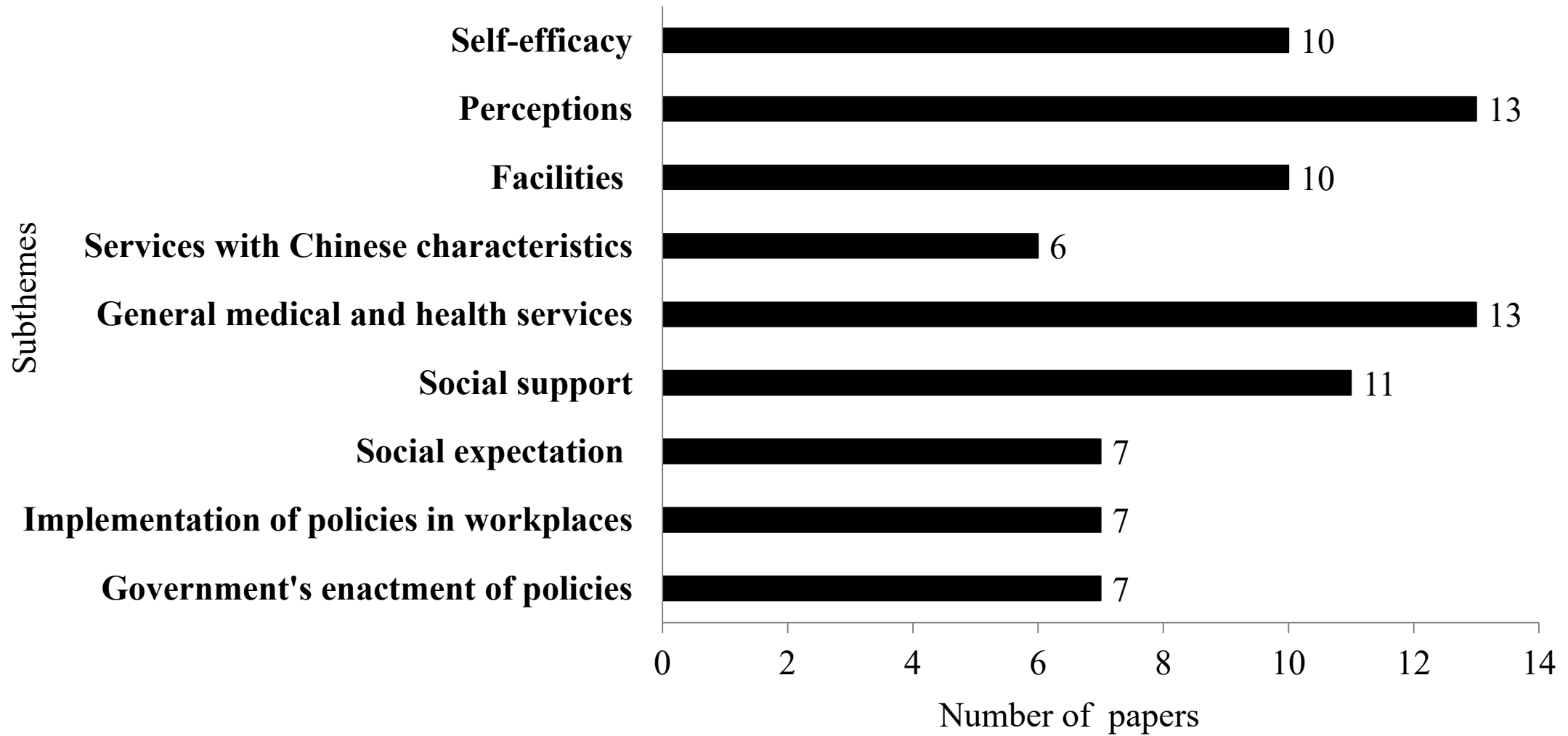

Supplement: Supplementary file 1 — Figure S1. Supporting Information [file MCN-17-e13251-s003.pdf]
