# Supplementary material for: Factors influencing breastfeeding practices in China: A meta‐aggregation of qualitative studies
Source: Matern Child Nutr. 2021 Aug 6;17(4):e13251. doi: 10.1111/mcn.13251 (PMC8476444; doi:10.1111/mcn.13251)
Supplement: Supplementary file 2 — Table S1 Search strategy [file MCN-17-e13251-s004.docx]

Supplementary table 1 Search strategy

| Concept 1:  **Greater China Region** |  | Concept 2:  **Breastfeeding** |
| --- | --- | --- |
| China *OR* | AND | Breastfeeding (breast feed, breast-feed, feed, feeding breast feed ) *OR* |
| Chinese *OR* |  | Maternal milk (human milk) *OR* |
| Taiwan *OR* |  | Lactation *OR* |
| Taiwanese OR |  | Infant feeding OR |
| Hong Kong *OR* |  | Mother health (maternal health) *OR* |
| Macao OR |  | Nursing |
| Macanese |  |  |
