# Supplementary material for: Factors influencing breastfeeding practices in China: A meta‐aggregation of qualitative studies
Source: Matern Child Nutr. 2021 Aug 6;17(4):e13251. doi: 10.1111/mcn.13251 (PMC8476444; doi:10.1111/mcn.13251)
Supplement: Supplementary file 3 — Table S2 Summary of CASP assessment of the included papers a [file MCN-17-e13251-s005.docx]

Supplementary table 2 Summary of CASP assessment of the included papers ^a^

|  | **1. Was there a clear statement of the aims of the research?** | **2. Is a qualitative methodology approriate?** | **3. Was the research design appropriate to address the aims of the research?** | **4. Was the recruitment strategy appropriate to the aims of the research?** | **5. Was the data collected in a way that addressed the research issue?** | **6. Has the relationship between researcher and participants been adequately considered?** | **7. Have ethical issues been taken into consideration?** | **8. Was the data analysis sufficiently rigorous?** | **9. Is there a clear statement of findings?** | **10. How valuable is the research?** | **Total score** |
| --- | --- | --- | --- | --- | --- | --- | --- | --- | --- | --- | --- |
| **Chang, Rowe, & Goopy, 2014** | 2 | 2 | 2 | 2 | 1 | 1 | 2 | 2 | 2 | 2 | 18 |
| **Chen et al., 2019** | 2 | 2 | 2 | 2 | 0 | 1 | 2 | 2 | 2 | 2 | 17 |
| **Chen, Y., et al., 2016** | 2 | 2 | 2 | 2 | 2 | 1 | 2 | 2 | 2 | 2 | 19 |
| **Chen, Zhou, Zhang, & Sheng, 2016;** | 2 | 2 | 2 | 1 | 1 | 1 | 2 | 2 | 2 | 2 | 17 |
| **Hanser & Li, 2017** | 2 | 2 | 2 | 1 | 2 | 1 | 2 | 2 | 2 | 2 | 18 |
| **Ho & McGrath, 2011** | 2 | 2 | 0 | 1 | 1 | 1 | 2 | 2 | 1 | 2 | 14 |
| **Hu et al., 2013** | 2 | 2 | 2 | 2 | 2 | 1 | 2 | 2 | 2 | 2 | 19 |
| **Jiang et al., 2012** | 2 | 2 | 2 | 2 | 2 | 1 | 2 | 2 | 2 | 2 | 19 |
| **Li, Yi, Liu, Luo, & Shen, 2014** | 2 | 2 | 2 | 2 | 2 | 1 | 2 | 0 | 1 | 2 | 16 |
| **Ouyang, Su, & Redding, 2016** | 2 | 2 | 2 | 2 | 1 | 1 | 2 | 2 | 2 | 2 | 18 |
| **Tarrant, Dodgson, & Wu, 2014** | 2 | 2 | 2 | 2 | 2 | 1 | 2 | 2 | 2 | 2 | 19 |
| **Wu, Kuo, & Lin, 2008** | 2 | 2 | 2 | 2 | 2 | 1 | 2 | 2 | 2 | 2 | 19 |
| **Wu, Yang, Zhang, & Cai, 2017** | 2 | 2 | 2 | 2 | 2 | 1 | 2 | 2 | 2 | 2 | 19 |
| **Yan, Peng, & Zhou, 2018** | 2 | 2 | 2 | 1 | 2 | 1 | 2 | 2 | 2 | 2 | 18 |
| **Yang, Jiang, Li, & Qian, 2011** | 2 | 2 | 2 | 2 | 1 | 1 | 2 | 2 | 2 | 2 | 18 |
| **Yang et al., 2015** | 2 | 2 | 2 | 1 | 2 | 1 | 2 | 2 | 2 | 2 | 18 |
| **Yang, Liu, Yi, Zhou, & Wan, 2016** | 2 | 2 | 2 | 2 | 1 | 1 | 2 | 2 | 1 | 2 | 17 |
| **Yu, Liu, Kong, & Dai, 2018** | 2 | 2 | 2 | 1 | 2 | 1 | 2 | 2 | 2 | 2 | 18 |
| **Yu, Wu, & Zhuang, 2013** | 2 | 2 | 2 | 2 | 2 | 1 | 2 | 2 | 2 | 2 | 19 |
| **Zhang et al., 2015** | 2 | 2 | 2 | 1 | 1 | 1 | 2 | 2 | 2 | 2 | 17 |
| **Zhang, Jin, Vereijken, Stahl, & Jiang, 2018** | 2 | 2 | 2 | 2 | 2 | 2 | 2 | 2 | 2 | 2 | 20 |
| **Zhao, Ouyang, & Redding, 2018** | 2 | 2 | 2 | 2 | 2 | 1 | 2 | 2 | 2 | 2 | 19 |

1. Assessed using the 2018 Critical Appraisal Skills Programme (CASP) checklist. Numerical values were assigned to the three possible answers to the CASP questions (yes=2, can’t tell=1, no=0),
