# Supplementary material for: Factors influencing breastfeeding practices in China: A meta‐aggregation of qualitative studies
Source: Matern Child Nutr. 2021 Aug 6;17(4):e13251. doi: 10.1111/mcn.13251 (PMC8476444; doi:10.1111/mcn.13251)
Supplement: Supplementary file 4 — Table S3a Full thematic analysis [file MCN-17-e13251-s002.docx]

Supplementary table 3a Full thematic analysis

| Themes | Subthemes | Quotes |
| --- | --- | --- |
| Individual | Perceptions |  |
|  |  | "You can see that reports about quality problems of formula continue to emerge, and the imported products are not exceptions. Although it is hard to breastfeed and go to work simultaneously, I still decide to give my child more breastfeeding." "A few years ago, TV and newspapers reported the quality problems of Sanlu formula. Some time later, some foreign milk powders were also reported to have quality problems. It is much safer to eat breast milk."(Li, J.P., et al., 2014) |
|  |  | "Breastfeeding is the best. It is safe. You know the ‘melamine infant formula contamination incidents in 2008,’ I worry about the quality of formula." (Jiang, H., et al., 2012) |
|  |  | "After the formula scandal in 2008, Chinese mothers are more willing to breastfeed their babies." (Zhang, Y., et al., 2018) |
|  |  | "Infant formula preference is often influenced by advertising for it everywhere." (Gao, H., et al., 2016) |
|  |  | "As long as the safety is assured, such as no melamine contamination, the infant formula has better nutrition."(Zhang, K., et al., 2015) |
|  |  | "I thought breastmilk is the optimal food for babies, which can provide better nutrient, so I will keep breastfeeding." （Yan, N., et al.，2018） |
|  |  | "If baby is fed by a mixed way, the nutrition would be better. I know foremilk would help baby’s immune system, but how about other nutrients? Formula has many nutritional elements. Furthermore, it would be easy for weaning." (Jiang, H., et al., 2012) |
|  |  | "I don’t think it (breast milk) is worthwhile (taida de yongchu). I think formula is just as good based on my son’s growth percentile." (Hanser, A., et al., 2017) |
|  |  | "After menstruation resumed, breastmilk would have no any nutrition value, just like water." (Jiang, H., et al., 2012) |
|  |  | "When my baby was 5 months old, my mother-in-law said breast milk is short of nutrient and offered to add formula. I was so upset. But after I had consulted the hospital, she didn’t insist any more." (Yu, G.L., et al., 2018) |
|  |  | "I think that after menstruation resumed, the breast milk would become not as nutritious as before. I heard from my mother." (Zhang, Y., et al., 2018) |
|  |  | "I know breastfeeding is good, but there may be a lack of nutrient? Therefore, I changed to feed my baby with formula instead of breast milk at the forth month." "Although breastfeeding is good, my baby is premature. It is not enough to breastfeed a premature infant? I fed my baby with formula predominately all the time, fear of breastmilk is inadequate in terms of nutrition." (Yang, H.J., et al., 2015) |
|  |  | "My breast milk looks like water, very thin. I think it's no longer nutritious." "My aunt said after 10 months, the breast milk would be similar to water and not nutritious enough for the baby." (Zhang, Y., et al., 2018) |
|  |  | "The volume of my breast milk was enough so that my baby couldn’t finish eating. However, when I expressed breast milk into the bottle, I found it diluted and with a light color. Breast milk like this must lack nutrient so I changed to feed formula." (Wu, Q.X., et al., 2017) |
|  |  | "Probably the nutrient content in my milk is not enough, therefore I use infant formula as supplement." " Because infant formula has many advantages, it is rich in some nutrients like DHA (docosahexaenoic acid), taurine that are good for baby’s development. But the content of these nutrients is low in mothers’ milk." "Many mothers believe that 'infant formula is necessary in addition to breast milk'." (Gao, H., et al., 2016) |
|  |  | "I was told by doctors at regular check-ups that if I breastfed fully, the baby should take vitamin A or vitamin D supplements. However, it is not necessary for formula-fed babies to consume these supplements. So the nutrients contained in formula are more comprehensive." "The infant formula is more beneficial, as it has many additional and essential vitamins and minerals. The types and quantities of nutrients are clearly labeled on the packaging."(Zhang, K., et al., 2015) |
|  |  | "Breastfeeding is inconvenient and exhausting so we supplemented a few meals of formula." "I heard of that there isn’t much nutrient in breast milk after 3 months postpartum so we began to supplement formula at the third month." "We didn’t practice breastfeeding. Many children who were fed with formula also grew well and we offered my baby with the most optimal formula, which isn’t inferior to breast milk." (Chen, Y., et al., 2016) |
|  |  | "My sister is formula fed only. Her baby gained weight more quickly than my daughter. It is because the nutrients in formula are better."(Zhang, K., et al., 2015) |
|  |  | "Although I know breastfeeding is good, but what’s the difference between breastmilk and infant formula? Formula includes many nutrients. Does breastmilk have enough [nutrients] too? I don’t know" (Jiang, H., et al., 2012) |
|  |  | "In fact, most of the mothers are willing to breastfeed their babies because breast milk is the best. But there is no way to breastfeed exclusively, because their breast milk is not enough." (Gao, H., et al., 2016) |
|  |  | "Many mothers never heard about exclusive breastfeeding." (Gao, H., et al., 2016) |
|  |  | "In my work experience, there were many mothers who initiated formula feeding at two or three months postpartum. The main reason was that their milk couldn’t satisfy the baby’s hunger as they found the baby got hungry very quickly, was unsettled or crying a lot."(Doctor from Maternal and Child Care Clinic)(Zhang, K., et al., 2015) |
|  |  | "After the child was born, I felt that my breast milk was not enough. I was worried that she did not have enough milk, so I gave her some milk. I stop lactating gradually."(Li, J.P., et al., 2014) |
|  |  | "My first baby ate every less than 2 hours, I fed him with formula instead of breast milk, worry about that my breast milk was not enough." (Wu, Q.X., et al., 2017) |
|  |  | "At the idea of that I had two babies, I felt that they didn’t have adequate nutrition, so I fed them with formula apart from breast milk." "Although I knew that breastfeeding is good, my brain went blank when I really faced them. I was always afraid that they were hungry, so I fed formula to them without breast milk." (Yang, H.J., et al., 2015) |
|  |  | "My baby suckled frequently, but I thought she did not eat much. When she cried, I felt very worried. I thought I didn't have enough milk, so I let my mother-in-law feed her some formula." (Zhang, Y., et al., 2018) |
|  |  | "My baby couldn’t sleep for a long time with blubbering for one hour or more, especially at night, so that we failed to fall asleep. We considered that it was blame for inadequate breast milk so we fed him with formula for supplement." (Chen, Y., et al., 2016) |
|  |  | "We know breast milk is the best, but we were afraid that the amount is not enough. We don’t know when the baby is full." "I am afraid that my breast milk is not enough and consequently my baby will not grow/develop properly." (Gao, H., et al., 2016) |
|  |  | "Because sometimes, if the breast milk is not enough, the baby will cry, so we give him infant formula in addition." "I have no idea. I want to breastfeed exclusively, but the amount of my breast milk is not enough." (Gao, H., et al., 2016) |
|  |  | "As we only have one child, all parents cherish their babies and worry too much that their baby may get hungry." "Everyone treasures the baby so much and thinks that with infant formula the baby will develop better." (Gao, H., et al., 2016) |
|  |  | "I had no experience. There was no one to help me. I don't know how much the baby had taken. I don't know if it is okay after feeding. The key point is I didn't know whether my baby was drinking or not." "There was a problem, as my breasts are not a feeding bottle or cup, I could see my baby's sucking but I didn't know how much my baby had taken. I doubted because I didn't know if my baby was full or not. Because the nurse told me that sucking didn't mean drinking." (Tarrant, M., et al., 2014) |
|  |  | "What they were concerned with the most was if the baby was full or not. They worried that the baby might not get enough food. They didn't ask me not to breastfeed. They asked me to supplement with formula milk after breastfeeding." (Tarrant, M., et al., 2014) |
|  |  | "I stopped breastfeeding when my baby was about three months of age. My breast milk started to dry up at that time. My baby often cried right after feeding." "My daughter was always crying and unsettled. She became hungry again in a short time after breastfeeding. She looked for food all the time. However, these situations disappeared when I began to give her formula."(Zhang, K., et al., 2015) |
|  |  | "I began to give my baby infant formula a few days after delivery. I never feel my breasts are full, even after several hours without feeding."(Zhang, K., et al., 2015) |
|  |  | "Before going to work, I considered switching from breast feeding to formula for my child gradually, because I was worried that my baby would not adapt to this change. But after consulting, I knew that I did not have to worry. Sucking was the nature of the baby... After going to work...I chose the method of expressing and storing milk."(Li, J.P., et al., 2014) |
|  |  | "One of my friends told me if I breastfeed exclusively, my baby may refuse the bottle when I want to wean. In order to help my baby getting used to bottle feeding, I give him some formula every day."(Zhang, K., et al., 2015) |
|  |  | "Breastfeeding is the best way to communicate emotionally between the mom and the baby. It is breastfeeding that bonds me with my baby. Especially when I was breastfeeding, I can feel I love my baby deeply."（Yan, N., et al.，2018） |
|  |  | "When my child is sick, gets hurt and pain, breastfeeding can always allow him to obtain comfort from his or her mom‘s arms so that he or she can recover quickly."（Yan, N., et al.，2018） |
|  |  | "I feel that breast milk is not just a kind of food (shiwu), but [breastfeeding] is also a kind of exchange of feeling (qinggan jiaoliu) between me and [my baby]." (Hanser, A., et al., 2017) |
|  |  | "Breastfeeding, a way to exhibit maternal love, can fulfill her the sense of worth and proud in the process of her baby growing."（Yan, N., et al.，2018） |
|  |  | "For a mother, you always want to give your baby the best, and because I feel that breast milk is the best, so I’ve decided to give him the best—breast milk." (Hanser, A., et al., 2017) |
|  |  | "I think because I didn’t adhere to breastfeeding my first child, his physical constitution is poor. I hope to breastfeed my second child."(Wu, Q.X., et al., 2017) |
|  |  | "I have read professional and unprofessional materials regarding breastfeeding, so I agree with breastfeeding and natural weaning. "（Yan, N., et al.，2018） |
|  |  | "Breastfeeding can also lower the risk of breast cancer and ovarian cancer and promote establishing the parenthood relationship."（Yan, N., et al.，2018） |
|  |  | ‘‘[Breastfeeding is] good for baby’s, especially the foremilk very good for baby’s immune system. In addition, breastfeeding could improve the mother–baby relationship." (Jiang, H., et al., 2012) |
|  |  | "Before childbirth, I thought if I could, I would breastfeed. It's instinct. And breast milk is the best gift for the baby." "I always believe in BF, and I will breastfeed if possible…I think that BF is safer and better for the baby's health."(Zhang, Y., et al., 2018) |
|  |  | "I certainly wanted to have my baby breastfed, which is healthy to him." "I really wanted to know the benefits for the baby and the mother of breastfeeding for the first time of being a father." (Chen, Y., et al., 2016) |
|  |  | "I breastfed exclusively and he was very thin. I was very worried because he weighed 7.5 pounds when he was born. But then he only weighed a bit more than six pounds.So I was afraid…. He cried because he's not getting enough. So I used formula milk as supplement. After I used formula milk as supplement, his weighted increased greatly." (Tarrant, M., et al., 2014) |
|  |  | "On the second day after childbirth, jaundice was tested and my baby was diagnosed with jaundice. So I gave my baby some water according to the advice from my mother-in-law." (Zhang, Y., et al., 2018) |
|  |  | "I learned of the difference between physiological jaundice and breastfeeding jaundice, how to observe the change of infant jaundice as well as when to see a doctor through explanation of breastfeeding at the outpatient." (Wu, Q.X., et al., 2017) |
|  |  | "My baby had a jaundice at the third day, which my relatives said was caused by breastfeeding. But I learned it didn’t happen like this after consulting the hospital." (Yu, G.L., et al., 2018) |
|  |  | "Mothers choose infant formula instead of breastfeeding for the shape of their breasts; it’s a common problem. Anyway, I don’t care about my figure, I will insist on breastfeeding." "At present, mothers have different perceptions. Some mothers do not want to breastfeed, just like my friend who believes breastfeeding is harmful for her figure." (Gao, H., et al., 2016) |
|  |  | "If you are a professional woman, you definitely want to keep your figure…but after a woman breastfeeds, her breasts shrink and sag." (Hanser, A., et al., 2017) |
|  |  | "I've long heard that breastfeeding can affect body recovery. If I were still such fat after back to work, it would ruin my image? Besides, breastfeeding would cause breast sagging, which I don't want to be like that. So the my baby started to eat formula very early." "I do not want to show up in my company with this appearance (She depicted her body shape and thought she was fat). I need to take measures early, so I had to give my child formula."(Li, J.P., et al., 2014) |
|  |  | "I thought when the baby was born, the breastmilk would come out naturally and the baby would just put her mouth to the nipple, then she could be fed. But it's totally a different thing. …I didn't know how to feed my baby." "It was much more difficult than I thought. … After I watched the video, I told myself that it is very easy to do. You think it's natural. Everyone can do it. But it's quite different." (Tarrant, M., et al., 2014) |
|  |  | "In fact, I did not learn about BF(breastfeeding) in detail. I just hoped to breastfeed by that time (during pregnancy) and assumed that everything would be fine, so I did not learn about what should be prepared." "They (pregnant women) do not expect difficulties in BF(breastfeeding), and they think breast milk will come naturally after childbirth. They do not realize that many problems may happen when starting BF(breastfeeding), which makes them easily give up." (Zhang, Y., et al., 2018) |
|  | Self-efficacy |  |
|  |  | "I think because I didn’t adhere to breastfeeding my first child, his physical constitution is poor. I hope to breastfeed my second child."(Wu, Q.X., et al., 2017) |
|  |  | "I was busy with working when I had my first child. When I was hospitalized, the nurses told me skills and notes of breastfeeding roughly. But I didn’t pay special attention to those because of pain in wounds and physical fatigue. A severe mastitis following fever came soon after I was discharged and I was treated with puncture and drainage for one month. It was so horrible! At the idea of breastfeeding again I was reminded of the remaining horror." (Wu, Q.X., et al., 2017) |
|  |  | "I think that milk supply is related to breast size. My breast size is small and I'm a little skinny, so I have been worried about low milk production."(Zhang, Y., et al., 2018) |
|  |  | "I had a hunch that my breast milk would be insufficient, because my mother did not produce enough breast milk."(Zhang, Y., et al., 2018) |
|  |  | "My mother-in-law insisted on adding water, considering that babies are short of water if only fed with breast milk. She said she did this when she raised her child before." (Yu, G.L., et al., 2018) |
|  |  | "My sister is formula fed only. Her baby gained weight more quickly than my daughter. It is because the nutrients in formula are better."(Zhang, K., et al., 2015) |
|  |  | "I bought infant formula during pregnancy, because other mothers all told me that the breast milk production would be low in the first few days after childbirth."(Zhang, Y., et al., 2018) |
|  |  | "At first, I didn’t know how to breastfeed and my baby didn’t suck nipples very well, either. At the sight of the mother next to me breastfeeding expertly, I was anxious for being unable to breastfeed. I was worried, guilty and thought I was not qualified [to be a mother]." (Yu, G.L., et al., 2018) |
|  |  | "I had no breast milk for the first several days after delivery. Although my milk supply is getting more and more, it is still not enough. My baby’s appetite is also becoming larger. Now she needs more than 100 mL per meal, but I can only express 90 mL."(Zhang, K., et al., 2015) |
|  |  | "I wanted to breastfeed so much at the time, but I had insufficient milk. And I had great pressure. My mother-in-law cared about me,but her caring gave me pressure. She asked me why I did not breastfeed my baby. But it wasn't that I did not want to breastfeed, it was just that my baby was not able to suck. I did try for many times, but in vain." (Tarrant, M., et al., 2014) |
|  |  | "Continue to breastfeed after work? It is impossible. The workload in the place I work is very heavy. I do not have time and energy." "I work for 9 hours daily on average and have heavy work stress. I had no time or energy to breatfeed." "Sometimes, after a whole day of work, I feel deadly tired. I do not want to eat anything and do not want to breastfeed my child." "I insisted on it for a while, but I felt that everyone was tired and gave up [breastfeeding after work]."(Li, J.P., et al., 2014) |
|  |  | "I primarily took care of my baby for four and a half months postpartum and I was very fatigue and couldn’t sleep well. Then I needed to work and was unable to attend to all these things so I had to give up breastfeeding."(Yang, H.J., et al., 2015) |
|  |  | "It is so hard for us to continue breastfeeding. ... I am very tired and sleepy at work because I have to get up several times at night to feed her whenever she cries. I still have to get up early every morning to go to work. .."(Chen, J.W., et al., 2019) |
|  |  | "I just felt that I didn’t have enough sleep because I could fall asleep when the baby was sleeping while I wanted to sleep when the baby began to cry." "I had always suffered sleeplessness all night long with no time to catch up on sleep at the daytime. With things going on like this for a long time, I was worn out and didn’t want to do anything. Also, I had a bad temper and want to burst into tears with no reasons. I didn’t know what to do." (Yu, G.L., et al., 2018) |
|  |  | "There was even no time for me to have a meal quietly after discharged."(Yu, G.L., et al., 2018) |
|  |  | "I was nervous in the fear of that the baby had even a bit of discomfort. I couldn’t sleep soundly, worried about that my baby would vomit milk or the quilt covered his nose." (Yu, G.L., et al., 2018) |
|  |  | "I held my baby when breastfeeding all the day including the night, resulting in pains in my waist and wrists and I was exhausted." (Yu, G.L., et al., 2018) |
|  |  | "During the first month postpartum, I felt very tired…I breastfed my baby once or twice every night, and sometimes I was too tired, then my mother-in-law just fed baby some formula." (Zhang, Y., et al., 2018) |
|  |  | "I just fell in sleep and then the baby cried for BF(breastfeeding), I had not slept well for the whole month, and I have experienced many troubles, including fever, breast block." (Zhang, Y., et al., 2018) |
|  |  | "The babies fed with infant formula will not be hungry easily. Their mothers do not need to wake up and feed the babies so frequently in the night. It’s more convenient." "Some mothers are using infant formula for convenience." (Gao, H., et al., 2016) |
|  |  | "I felt more comfortable because I didn't have to feed every two hours and other people could help me to feed the baby. Because when I breastfed, I had to wake up and I couldn't ask others to help. But for formula feeding, others can check the time for feeding, put the milk into the bottle and then they can feed the baby. That's it. I didn't get upset. I felt better not having to breastfeed." "The frequency of breast-feeding was high because I had to feed the baby whenever she cried. I felt more comfortable after I changed to feed with formula milk because she's full and she cried less often." (Tarrant, M., et al., 2014) |
|  |  | "I suffered from extreme sleep loss when my baby was under one month old. I had to breastfeed her every one or two hours, even at night. After starting to give her formula, I feel more relaxed. I can now have a good rest at night."(Zhang, K., et al., 2015) |
|  |  | "Many mothers give formula to help their baby sleep longer at night. The sleep-deprived new mothers are eager to catch up some sleep." (Nurse from Maternal and Child Care Clinic)(Zhang, K., et al., 2015) |
|  |  | "During sucking, the baby kicked me unconsciously, it was very painful and made me feel bad… I was so worried about the wound dehiscence." (Zhang, Y., et al., 2018) |
|  |  | "Because I had a Caesarean section, the wound was still painful after birth. Therefore, I started to breastfeed my baby on the third day after delivery." (Gao, H., et al., 2016) |
|  |  | "It’s painful after Caesarean section. It may influence exclusive breastfeeding [hard to hold the baby]." (Gao, H., et al., 2016) |
|  |  | "My baby started to teeth now, she learned to bite and nibble, and there was even a tooth-mark on my breast, therefore I really wanted to stop BF(breastfeeding) at that time." (Zhang, Y., et al., 2018) |
|  |  | "At that period pf time I was discouraged of that I couldn’t help with anything and I didn’t know how to do because there is the first time for us to be parents. I couldn’t sleep well at night. There had been several times when my wife' s breasts were plump or she suffered a fever so that we wanted to feed formula directly, reckoning that it might relieve us." (Chen, Y., et al., 2016) |
|  |  | "If I breastfeed, I need to control my diet, such as no spicy food and no cold food, but I like to eat those foods. Furthermore, I can't go outside too long, it's really annoying." (Zhang, Y., et al., 2018) |
|  |  | "Formula allows other people to feed the infant. After being confined home for a month after childbirth, some mothers would like their family members help with feeding so they can go outside shopping or visiting friends." (Nurse from Maternal and Child Care Clinic)(Zhang, K., et al., 2015) |

Supplementary table 2b Full thematic analysis

| Themes | Subthemes | Quotes |
| --- | --- | --- |
| Environment | General medical and health services |  |
|  |  | "I always have prenatal examination on time and attend school for pregnant women, so I get the knowledge of the benefits of breastfeeding." (Yang, H.J., et al., 2015) |
|  |  | "In the prenatal education, I knew exclusive breastfeeding should last for 6 months." (Jiang, H., et al., 2012) |
|  |  | "I met a very nice nurse. She kept reminding me of BF(breastfeeding) and checked the feeding condition every 2–3 hours." (Zhang, Y., et al., 2018) |
|  |  | "Then she (the nurse) taught me how to let baby suckle the nipple. She told me that the less the milk comes out, the more often you need to let the baby suckle. The reflex was to be established through this way." (Zhang, Y., et al., 2018) |
|  |  | "When I was hospitalized, the nurse recommended that I should not take the chicken soup or fish soup before I could lacate smoothly."(Yu, H., et al., 2013) |
|  |  | "In fact the hospital stay is really short. For example, in Shanghai after childbirth, mothers with normal vaginal delivery are discharged in 24 hours, while mothers with CS can stay for 2–3 days. So many mothers might have the BF problems at home." (A nurse) (Zhang, Y., et al., 2018) |
|  |  | "It was very difficult and I felt a lot of pain. My milk wasn't enough and he drank a lot. He seemed to be still hungry even if I had breastfed him for a long time.The nurse asked me if this was the second time feeding the baby. And I said I was still feeding him – I fed him continuously. So the nurse took the baby and gave him bottle milk in order to let me take a rest." (Tarrant, M., et al., 2014) |
|  |  | "No specific guidance on breastfeeding when I lived in hospital after childbirth. They (health staff) just told us not to bring bottle milk to the hospital. Every day, nurses asked me whether I had breastmilk. If I had not, she then gave us a cup with a fixed quantity of formula to feed the baby every 4 hours. They didn’t require me to breastfeed my baby and didn’t teach me how to breastfeed the baby." (Jiang, H., et al., 2012) |
|  |  | "The doctors and nurses didn’t tell us to start breastfeeding immediately [after childbirth]" (Gao, H., et al., 2016) |
|  |  | "In addition to assisting mothers with breastfeeding, we have many other duties to perform every day. There are too many mothers delivering babies but not enough doctors, nurses or midwives. Sometimes when we are extremely busy, it is impossible to give every mother step-by-step instructions on breast feeding." (Nurse from Gynecology and Obstetrics Department)(Zhang, K., et al., 2015) |
|  |  | "In hospital, the nurse was busy and just told me to let baby suckle more." (Zhang, Y., et al., 2018) |
|  |  | "I am too busy, and the workload is heavy. I am in charge of 10 patients [at the same time], and there are so many things I need to care about …I need to instruct them the position of BF and the basic knowledge of baby care. Sometimes I could not tell her in details, especially at night, there are only two nurses [in total including her]." (A midwife) "The problem is that our doctors have little time to teach and guide mothers about the detailed [breastfeeding] knowledge. In general, doctors are busy in diagnosing and treatment." (A researcher) (Zhang, Y., et al., 2018) |
|  |  | "I was admitted at 3 pm, and was discharged the next noon. I had no time to communicate with doctors or nurses. And my breast milk still did not come when I left the hospital." "I had a CS(caesarean section), and my breast milk came on the 3rd day, the amount was really little. The nurse told me to let baby suckle more, I did, but it didn't work. When I was discharged [on the 4th day], the problem still wasn't solved." (Zhang, Y., et al., 2018) |
|  |  | "They didn't teach me.They just gave the baby to me after six hours, and then said nothing.They just said,'You can try.'…Because the nurse was quite busy." "Because of insufficient nurses,they always asked me to try by myself when I asked for help. I couldn't help because after I tried, I didn't know what to do." (Tarrant, M., et al., 2014) |
|  |  | "Equipped with knowledge learned from breastfeeding outpatient, I have ample confidence in breastfeeding my second baby." "Now breastfeeding outpatient teaches me how to judge whether babies are full via models and evidence-based cases, so I am confident in doing better this time." (Wu, Q.X., et al., 2017) |
|  |  | "The breastfeeding outpatient made me learn a great amount of breastfeeding skills." "I was suddenly enlightened by the breastfeeding outpatient, because it allowed me to understand what I didn’t before. Besides, illustrations hung on the wall of the outpatient which are easy to understand, let me know the ways I expressed breast milk and massaged breasts before are wrong. The breastfeeding outpatient is a good assistant for lactation mums." "Massages, physical therapies and methods of frequent breastfeeding, right postures of sucking nipples provided by the breastfeeding outpatient relieved my pressure of breastfeeding this baby." (Wu, Q.X., et al., 2017) |
|  |  | "I didn't know many of my perceptions were wrong until I finished the scale. I learned a lot of proffesional knowledge from the assessment scale." "I attained a high score in assessment by Breastfeeding Confidence Scale[conducted by the breastfeeding outpatient], so I am full of confidence and sure that I can manage to conduct breastfeeding." (Wu, Q.X., et al., 2017) |
|  |  | "I learned about how to continue breastfeeding after back to work online and by telephone consultation when I was about to return to work. Sometimes when I took my child to have preventive injections, I also inquiried about related knowledge in the child health clinic. I think I have alreaby become an expert in this field."(Li, J.P., et al., 2014) |
|  |  | "When my baby was 5 months old, my mother-in-law said breast milk is short of nutrient and offered to add formula. I was so upset. But after I had consulted the hospital, she didn’t insist any more." (Yu, G.L., et al., 2018) |
|  |  | "This outpatient clinic is good. I had a lot questions before, and I searched on the internet. There were so many different answers, some were even contrary. I did not know what to do on earth. When I took my baby for disease screening, I heard about this outpatient clinic(breastfeeding outpatient) and I came here. The nurse there gave me detailed answers. I was quite satisfied with this clinic service." "Nowadays, the information is well developed, but I was not sure whether they are reliable. My husband said we should make some consultations here, as this is a hospital, the words doctors and nurses said were scientific."(Yu, H., et al., 2013) |
|  |  | " I had been discharged from the hospital for half a month, but the volumn of breast milk I could lacate was still small... After consultation[in the breastfeeding outpatient], I knew that I could have these soup properly 4 to 5 days after delivey when the breast tube could pipe smoothly. What's more, I should eat both animal and vegetable foods."(Yu, H., et al., 2013) |
|  |  | "My breast swelled painfully...I felt sad and I wanted to give up breastfeeding and to use formula. After coming here(breastfeeding outpatient), the nurse did massage for me... The nurse said, 'Hold on, you are a brave mother.' She also taught me how to do it. Finally, she used a breast pump to help suck for a while...The milk was unblocked and the baby was satisfied with the food." "I did massage at home, but the effect was not good...Then I came to do consultation[in the breastfeeding outpatient], the nurse asked me the way I did massage. Only then did I know that it would be effective to massage from the root until the nuggets became soft...Now the babies are eating well."(Yu, H., et al., 2013) |
|  |  | "After maternity leave, I will go back to work. I was worried that my baby would refused to take infant formula if I breastfeed him at present... The consulting nurse[in the breastfeeding outpatient] told me to continue breastfeeding during maternity leave. She suggested me that after returning to work, I could squeeze breast milk in advance, store it in the fridge, and warm it before giving it to my baby. This was still a kind of breastfeeding. I did not know breast milk could be frozen before."(Yu, H., et al., 2013) |
|  |  | "Before going to work, I considered switching from breastfeeding to formula for my child gradually, because I was worried that my baby would not adapt to this change. But after consulting, I knew that I did not have to worry...After going to work, I could not breastfeed directly. I chose the method of expressing and storing milk."(Li, J.P., et al., 2014) |
|  |  | "There were many questions concerning breastfeeding. I would make calls to the hospital for consulting about what still couldn’t be solved by my relatives and friends. I wish hospitals can conduct home follow-up visits and establish a department of professional counseling to facilitate relevant problems solved." (Yu, G.L., et al., 2018) |
|  |  | "At the kid health check-up, doctors just asked me whether my baby was having breastmilk or formula. They didn’t say any others." (Jiang, H., et al., 2012) |
|  |  | "No any health staff member told me how to deal with the insufficient breastmilk production. How can I produce enough breastmilk?"(Jiang, H., et al., 2012) |
|  |  | "The community doctor came to my home and...then the doctor just asked me what kind of feeding I was having, but said nothing about BF(breastfeeding)." "The pediatrician asked me what kind of feeding method I used, BF or formula feeding. But he didn't give me any feedback." "The supporting system [for breastfeeding] needs to be improved. Pediatricians do not have time to talk to them (parents) [about breastfeeding]. And community doctors generally lack the knowledge about specific BF problems." (A policy maker) (Zhang, Y., et al., 2018) |
|  |  | "The guidance of breastfeeding given by postpartum obstetric hospital is limited, and the number and time of postpartum visits are limited. Community doctors only inquire about the way of feeding, but do not give more guidance to breastfeeding during children's physical examination."(Yang, D.L., et al., 2011) |
|  |  | "General practitioner suggested us to supplement formula concluding that my baby was underweight when my baby had a physical examination so we followed it." (Chen, Y., et al., 2016) |
|  |  | "We certainly needed guidance from professionals because it is our first time to be parents with no experience before." "I hoped to learn more about questions to be confronted with us and solutions of them during breastfeeding [especially about what I could do]." (Chen, Y., et al., 2016) |
|  |  | "General practitioners suggested to supplement formula for a slow gain of my baby’s body weight. However, medical staff of obstetrics said that we should make the baby eat more breast milk and less formula during telephone follow-ups. We didn’t know how to do." "We couldn’t settle with difficulties confronting us during breastfeeding, which warrants more professional guidance." (Chen, Y., et al., 2016) |
|  | Services with Chinese characteristics |  |
|  |  | "I hired a Kainaishi, she helped my lactation … she covered my breasts with some cabbage leaves, and massaged my breasts. And she said, you must empty breasts, otherwise you would have breast block." (Zhang, Y., et al., 2018) |
|  |  | "My Yuesao came on the 5th day, and before that I fed the baby formula at night. She forced the baby to suckle at night, even though the baby cried for 3 days, but I felt relieved when she (Yuesao) was there." (Zhang, Y., et al., 2018) |
|  |  | "When I was hospitalized, Yuesao asked me to carry feeding-bottles and formula. After my baby was born, she said it was good to adjust the baby to feeding-bottles now because you would feed him with feeding-bottles sooner or later when you went back to work." (Yu, G.L., et al., 2018) |
|  |  | "I think many Yuesao don't really understand BF, at least half [don't know]. They (Yuesao) feel convenient to feed infant with formula. Babies would have longer feeding intervals. They feed baby [infant formula] every four hours. At night, feed every five or six hours, so they can sleep more." (Zhang, Y., et al., 2018) |
|  |  | "Yuesao suggested me to feed my baby with some infant formula in the evening, so that he can have a longer sleep at night."(Zhang, K., et al., 2015) |
|  |  | "Breasetfeeding outpatients should be publicized more.You can provide scientific and profeesional services. I hope they are promoted more widely, in case that puerpera are misled by Yuesao and Cuirushi."(Wu, Q.X., et al., 2017) |
|  |  | "If you are not sure whether what you are doing is right or wrong, you can ask them(nurses)[ In-centre post-partum service and care] at any time, to make sure it is all right. They know how to give you verbal or behavioural support... at least I felt more emotional support."(Chang, S.-m., et al., 2013) |
|  |  | "I can get more breastfeeding resources in the centre[ In-centre post-partum service and care]."(Chang, S.-m., et al., 2013) |
|  | Facilities | "Usually I know [breastfeeding] from the Internet and one book. I was encouraged and decided to breastfeed by one book." (Jiang, H., et al., 2012) |
|  |  | "I was worried about someone interrupting me when I breast-feed my baby. A single room is better. But I don’t know where and how to find it. I have never seen a lactation room in the public places. Have you seen it?" (Zhao, Y., et al., 2018) |
|  |  | "It’s difficult to find a place to breastfeed outside home. I prefer to give my baby a bottle feed when I am down the street. Breastfeeding in public makes me feel uncomfortable."(Zhang, K., et al., 2015) |
|  |  | "There is a mummy room especially prepared for moms expressing breast milk at my work site."（Yan, N., et al.，2018） |
|  |  | "There are sofas, desks and a refrigerator in the room for expressing breast milk so that breast milk can be refrigerated in time." "The refrigerator at my work site can be used to store breast milk."（Yan, N., et al.，2018） |
|  |  | "The interruptions made me very uncomfortable. I felt nervous while I was expressing. However, this place was convenient for me, and it was not too far. I could save time." (Wu, C.H., et al., 2008) |
|  |  | " I have met many mothers who planned to continue breastfeeding after going back to work. But there are too many barriers to breast feeding at the workplace. For example, some have to express milk in toilets, and some hide under the office desk when pumping milk. A few workplaces such as hospitals are convenient for mothers to express milk while at work. But I believe most employers don’t provide any lactation facilities." "Expressing milk is a good way to continue breastfeeding. However, some mothers are too busy to express milk at work and many places simply don’t have a private room." (Doctor from Maternal and Child Care Clinic)"(Zhang, K., et al., 2015) |
|  |  | "A shortage of a private space for breastfeeding or expressing breast milk is the most inconvenient. What renders it so inconvenient to express breast milk in the office is that I had to lock the door in case of someone coming in for work stuff." "There is no specialized place for expressing and storing breast milk."（Yan, N., et al.，2018） |
|  |  | "We don't have a lactation room in the company. I have to express breast milk in a fitting room. And I put my breast milk in the public fridge. My male colleagues laughed at me. It is really embarrassing, and BF(breastfeeding) is impossible for working mothers" (Zhang, Y., et al., 2018) |
|  |  | "It is even hard to find a suitable place to squeeze and store breast milk in my work place." "Breastfeeding in my company is difficult. My company did not provide a private place for breastfeeding. And no collegues breastfeed at work." "It would be very considerable to have a nursing room in my company, however, this is an impractical idea, as my company is not likely to have such humanistic care at present."(Li, J.P., et al., 2014) |
|  |  | "There is no private room for pumping milk at my workplace. I don’t want to express my milk in the toilet, it is filthy. Therefore I go home to breastfeed during lunch time and of course after work." "It is challenging to find time to express milk whilst working, also not easy to find a place to express milk." "I will then stop breastfeeding totally. I have no other choice. My workplace is far from where I live and there is no breast feeding room and no refrigerator. It will be too difficult to continue breastfeeding."(Zhang, K., et al., 2015) |
|  |  | "It (a well facilitated lactation room) will at least provide me a private space where I can pump without rush. Now I can only pump in the bathroom, which is very dirty. What’s worse is that I have to rush because people are waiting outside. .." "Without a refrigerator, the storage and preservation of breast milk is also a big problem." "You have to go to the bathroom to pump, where there usually are no power outlets to charge the electric breast pump. .. It is very stuffy in the bathroom. .. In addition, that place gets me uncomfortable. .. makes me feel bad. .." (Chen, J.W., et al., 2019) |
|  |  | "I could wash my hands after I had touched the door knob, because there was no washing sink in the changing room. Thus, I brought a cup of water with me to clean my breasts before expressing milk." (Wu, C.H., et al., 2008) |

Supplementary table 2c Full thematic analysis

| Themes | Subthemes | Quotes |
| --- | --- | --- |
| Social network | Social expectation |  |
|  |  | "Many mothers wish to continue breastfeeding after they return to work. However, the breastfeeding support from employers was far from desirable."(Hospital staff )(Zhang, K., et al., 2015) |
|  |  | "Sometimes the child will cry in my work place, posing a bad influence. The manager took a dim view on this and suggested that my work should not be affected because of breastfeeding. Finally, I decided to squeeze the milk during working hours and bring it back after work."(Li, J.P., et al., 2014) |
|  |  | "When I went to express milk, my boss sometimes was uncomfortable with it, because it took half an hour every time. It seemed that I left my seat while I went to express milk. And some colleagues reported to the boss. …. My boss is male, and he is not empathetic." (Zhang, Y., et al., 2018) |
|  |  | "…I have to express breast milk in a fitting room. And I put my breast milk in the public fridge. My male colleagues laughed at me. It is really embarrassing, and BF(breastfeeding) is impossible for working mothers." (Zhang, Y., et al., 2018) |
|  |  | "Most colleagues in my company are in favor of breastfeeding and offered me a plenty of advices on breastfeeding." "Most colleagues in my company naturally breastfed their babies until back to work, so they encouraged me to breastfeed."（Yan, N., et al.，2018） |
|  |  | "My colleagues said that if I stop breastfeeding, I will be teased by them. They will say 'Why aren't you earnest?' and they will blame me continuously."(Chang, S.-m., et al., 2013) |
|  |  | "The majority of colleagues in my work unit insisted on breastfeeding and would understand and support each other. They encouraged me to breastfeed when I went back to work and sometimes helped me with work stuff. Because every woman are about to experience this period, so all of us will mutually understand."（Yan, N., et al.，2018） |
|  |  | "My colleagues and I are quite close, so my male colleagues would leave the room when I pumped. They were very nice". (Chen, J.W., et al., 2019) |
|  |  | "I could make an estimaton that there were not too many things to do in the coming half hour and asked co-workers to take care of patients for me. Sometimes it still depended upom who you worked with. Some of the nurses were not friendly about my expressing milk. Some times I felt guilty to ask for help" (Wu, C.H., et al., 2008) |
|  |  | "A person with high quality will be conscious of their image … [breast-feeding in public places] is related to a person’s quality and personality. Maybe my opinions are a little traditional and my thought is conservative, so I think it (breast-feeding in public places) should be a private behaviour. I don’t want others think that I am inelegant. It is the respect for others. After all, not all people can accept this behaviour." (Zhao, Y., et al., 2018) |
|  |  | "Since I was a child, I was told that exposing breasts in front of others was indecent. Of course, I think in public places lactation women should use something to cover or hide their breasts. Even so, I think that women had better not breast-feed in public places." (Zhao, Y., et al., 2018) |
|  |  | "I hope the public can understand that breast-feeding is normal and natural. It is not an uncivilized thing. So please eliminate discrimination and give us some support. I try not to bother others, also hope that other people don’t point fingers at me." (Zhao, Y., et al., 2018) |
|  |  | "What do people think when I feed the baby in front of them?" "Even if I use a nursing cloth to cover, people still know what I'm doing " (Zhang, Y., et al., 2018) |
|  |  | "Someone stare at me with unusual facial expression… I don’t want others to think that I am inelegant … so I try to avoid breast-feeding in public places" "I’ve never met anyone who’s been telling me what to do when I breast-fed in public places, but some looked at me strangely. I think they might believe that I’m morally deficient." (Zhao, Y., et al., 2018) |
|  | Social support |  |
|  |  | "My husband was very supportive to me and helped me to collect a lot of knowledge from the Internet, which made me more confident in continuing breastfeeding after returning to the work."(Li, J.P., et al., 2014) |
|  |  | "My husband is in favor of breastfeeding and thinks that it’s good for the baby’s health and my postnatal recovery. He would search the Internet for relevant knowledge." "At first, I was not willing to breastfeed because I have many concerns. My husband told me the benefits of breastfeeding obtained from 'Mummy lessons' and the Internet, and I accepted breastfeeding then." (Yang, H.J., et al., 2016) |
|  |  | "My husband was very busy, but he still got round to accompany me to take lessons of breastfeeding." (Yang, H.J., et al., 2016) |
|  |  | "I suffered caesarean section. After I went back to my patient ward after surgery, the nurse taught my husband how to hold my baby and how to let him drink breast milk. He (My husband)mastered these quickly and helped me [feed the baby breast milk] after my discharge." "When I was in hospital, the elder mainly took charge of my diet and he (my husband) helped me breastfeed." (Yang, H.J., et al., 2016) |
|  |  | "After four months postpartum, I went back work with great toil. My husband took care of the baby all the night and wouldn’t wake me up until it was time for breastfeeding, therefore I managed to keep breastfeeding." (Yang, H.J., et al., 2016) |
|  |  | "My husband often accompanied me to attend school for pregnant women during pregnancy. During lactation period, he agreed with me on everything in case of the impact of my bad mood on breast milk secretion." (Yu, G.L., et al., 2018) |
|  |  | " When I suffered an acute mastitis with great pain and fever during the first month postpartum, I wanted to cease breastfeeding. However, my husband positively contacted the hospital and helped me learn how to express breast milk at that time. Therefore, I keep breastfeeding and didn’t begin introduction of supplementary food until the sixth month." (Yang, H.J., et al., 2016) |
|  |  | "When my baby was 3 months old, we returned to the hometown, Although the journey took more than 10 hours, my husband found a suitable place for breastfeeding instead of feeding formula." (Yang, H.J., et al., 2016) |
|  |  | "He (My husband) went to other places for work when my baby was 1 month old, but he made telephone calls, sent photos via wechat every day. He also comforted me when I was in bad mood and made complaints occasionally." "When I was in a mood on account of conflicts with my mother-in-law over the diet during the first month after delivery, my husband got round to accompany and make me happy." "There was a period of time when I was blue with thought of bursting into tears and losing my temper, he(my husband) always tolerated me... Until now, I realized that I tended towards postpartum depression and I was thankful for his tolerance of me." (Yang, H.J., et al., 2016) |
|  |  | "Breastfeeding is a good thing so I am sure to support her and don’t let her feel too tired." "I accompanied her to make it through when we were faced with difficulties." "We decided to conduct breastfeeding, considering breastfeeding is good. Sometimes when we were confronted with difficulties, I would cheer her up so that we made it through." "I knew that how my wife longed for breastfeeding, so I keep encouraging her and gave her confidence. No matter what dilemma we had been in, we made it through." "After for running in 3 weeks, at the sight of my wife deftly breastfeeding the baby who had a good appetite, I couldn’t stop telling her ' You are the greatest mother in the world. Without eating breast milk he wouldn’t have become so chubby.'" (Chen, Y., et al., 2016) |
|  |  | "Now I do most of the housework instead of her to save her strength and have more energy to breastfeed without distraction. For breastfeeding is beneficial to both the mother and the baby, I must do my best to support it." (Chen, Y., et al., 2016) |
|  |  | "I pump the milk out and my husband drives to my office to pick it up every day.." (Chen, J.W., et al., 2019) |
|  |  | "My family did not help me. My husband could only pass me the bottle and the towel; he could do nothing else.They did not really care about whether I could breastfeed or not because they were mainly focused on the baby. " (Tarrant, M., et al., 2014) |
|  |  | "Both my husband and mother-in-law suggested me to breastfeed for a longer time, which freed me from concerns and considerations." "My mother-in-law cooked all kinds of soup for me every day and urged me to drink more. My husband wrote down which food are appropriate for me to eat and which are not, put them in the kitchen and let me to sleep as long as I had time." "My family members supported me to breastfeed and paid attention to my nutritional status." (Yu, G.L., et al., 2018) |
|  |  | "The wound was painful. And there was repeated failure of latching by the baby and cracked nipples. Everything seemed to be bad, and I want to give up. But my mother persuaded me to hold on and to feed the baby breast milk as much as possible." (Zhang, Y., et al., 2018) |
|  |  | "My mother-in-law insisted on adding water, considering that babies are short of water if only fed with breast milk. She said she did this when she raised her child before." (Yu, G.L., et al., 2018) |
|  |  | "I had a hunch that my breast milk would be insufficient, because my mother did not produce enough breast milk."(Zhang, Y., et al., 2018) |
|  |  | "My mother-in-law, who comes from rural areas, reckons that babies will develop better feeding on formula."(Yu, G.L., et al., 2018) |
|  |  | "On the second day after childbirth, jaundice was tested and my baby was diagnosed with jaundice. So I gave my baby some water according to the advice from my mother-in-law." (Zhang, Y., et al., 2018) |
|  |  | "My mother-in-law fed the baby formula secretly as she worried about the increase of jaundice of my baby and believed that only taking food could help." (Zhang, Y., et al., 2018) |
|  |  | "When my baby was 5 months old, my mother-in-law said breast milk is short of nutrient and offered to add formula. I was so upset. But after I had consulted the hospital, she didn’t insist any more." (Yu, G.L., et al., 2018) |
|  |  | "I think that after menstruation resumed, the breast milk would become not as nutritious as before. I heard from my mother" (Zhang, Y., et al., 2018) |
|  |  | " My mother-in-law did not breastfeed herself. When I did not have enough breastmilk or the baby cried, she would ask me to give the baby formula milk. So there was not much support from the family." (Tarrant, M., et al., 2014) |
|  |  | "My family thought that it is inconvenient and tiring to breastfeed while I hold a such a busy work. My mum said that formula fed children also grew up well. After listening them taking about this a lot, I gradually gave up breastfeeding when I was tierd."(Li, J.P., et al., 2014) |
|  |  | "Most families still have only one child in China. All the grandparents focus on the little baby after birth. Whenever the baby cries, the grandmothers and grandfathers become concerned that the baby remains hungry with breast milk. On the contrary, if the baby is formula fed, the grandparents usually feel more comfortable because they are able to see how much the baby has consumed. "(Nurse from Gynecology and Obstetrics Department)(Zhang, K., et al., 2015) |
|  |  | "My baby tended to fall asleep quickly at the breast. My mother-in-law therefore always said my baby wasn’t getting enough breast milk and needed to be fed with formula. Gradually, I became doubtful of my milk supply. Now I am topping up with formula after breastfeeding."(Zhang, K., et al., 2015) |
|  |  | "Both my mother and mother-in-law think my baby is too small compared to other babies. They often say, for example, the neighbor’s baby weighed already 8 kg at 4 months but my baby who is a few days older weighed less. My mum then asked me to eat more food and give my baby some formula."(Zhang, K., et al., 2015) |
|  |  | "If the baby was hungry, his grandfather would take him to the breakfast stand so that I could breastfeed him. ..." (Chen, J.W., et al., 2019) |
|  |  | "BF should last for last 10 months, at most 1 year. …. My friends all do so." (Zhang, Y., et al., 2018) |
|  |  | "Someone with experience told us water is necessary besides breast milk." (Gao, H., et al., 2016) |
|  |  | "Many of my friends in my generation didn’t have breast milk after childbirth at all." (Gao, H., et al., 2016) |
|  |  | "The feeling that 'all the other babies are fed with infant formula' makes mothers think it is wrong if they don’t give infant formula to their own babies. Mothers are afraid of the development of their babies falling behind others." (Gao, H., et al., 2016) |
|  |  | "I have a collegue, and we are friends at present.She continued breastfeeding long time after starting work, and she said all problems could be overcome once more prepartion were made. I think this has a profound effect on me."(Li, J.P., et al., 2014) |
|  |  | "Almost all my friends and college terminated breastfeeding after back to work, and their children grew well." "Two of my best friends stop breastfeeding after returning to work, and I also think it is difficult to work and breastfeed simultaneouly. So I weaned soon after back to work."(Li, J.P., et al., 2014) |
|  |  | "One of my friends told me if I breastfeed exclusively, my baby may refuse the bottle when I want to wean. In order to help my baby get used to bottle feeding, I give him some formula every day."(Zhang, K., et al., 2015) |
|  |  | "Many questions are similar, like breastfeeding questions. Many discussion and articles tell you how to breastfeed. So, I like to surf the Internet and I am used to it."(Chang, S.-m., et al., 2013) |
|  |  | "We just lack others’ consolation, others’ support. [The websites say] 'You should maintain breastfeeding, it is very good!'...I receive encouragement from there. Some positive things come out and beat the negative thoughts."(Chang, S.-m., et al., 2013) |
|  |  | "Actually mothers are getting their information more often through the internet. For example, they obtain knowledge on feeding methods from peers by joining some QQ groups. They may also exchange their feeding experience with other mothers via the group chat on We Chat." (Doctor from Maternal and Child Care Clinic)(Zhang, K., et al., 2015) |

Supplementary table 2d Full thematic analysis

| Themes | Subthemes | Quotes |
| --- | --- | --- |
| Policy | Government’s enactment |  |
|  |  | "Employers are legally required to give breastfeeding mothers one hour to breastfeed. But I think it is of little help. Mothers can seldom bring their baby to work. If the mother needs to go home to breastfeed, one hour is obviously not enough. Expressing milk is a good way to continue breastfeeding."(Doctor from Maternal and Child Care Clinic)(Zhang, K., et al., 2015) |
|  |  | "There is one hour in total for breastfeeding breaks, half an hour in the morning and half an hour in the afternoon. ... It is enough for me. … I drive home to breastfeed my baby every day." (Chen, J.W., et al., 2019) |
|  |  | "According to the provision of our country law, the maternity leave is 6 months, however, I had too much work on my hands and other people had difficulty in taking over my work. So I had to return to work earlier."(Li, J.P., et al., 2014) |
|  |  | "We cannot possibly be like rural people, nursing the child all along, we have to go to work." (Hanser, A., et al., 2017) |
|  |  | "I use the breastfeeding leave for early work leave". "One-hour leave for breastfeeding made little contributions to improving breastfeeding, as it usually took more than 2 hours to get to and from the workplace, so it is impossible to go home to feed the children at noon."(HU, Q.Z., et al., 2013) |
|  |  | "It is impossible to let my family take the child to my work place every time. My husband also has a job and could not take any time off. My house is far from work place, and the people taking care of my child is old, it is inconvenient to travel between my work place and my house." "My company allows two 30-min breastfeeding leave each day. However, it takes 30 mins from my work place to my home and it takes 1 hour to go back home and forth. This rule is very good, but it is not suitable for me."(Li, J.P., et al., 2014) |
|  |  | "I don’t think of one hour breastfeeding break as particularly useful nowadays for working mothers, but it is better than nothing. … I have to travel for about an hour from work to home, and so I am actually not able to feed my child during that only one-hour break time. ... But my colleagues and I can use that break to pump sometimes." "There is an hour of breastfeeding break for us, but it is a long commute between home and workplace. Going back and forth, it can add up to three to four hours. ... People in the unit would not let me just leave if there is so much work to do." (Chen, J.W., et al., 2019) |
|  |  | "It is so hard for us to continue breastfeeding. ... I am very tired and sleepy at work because I have to get up several times at night to feed her whenever she cries. I still have to get up early every morning to go to work. ... I wish I can have a longer maternity leave."(Chen, J.W., et al., 2019) |
|  |  | "Most families still have only one child in China. All the grandparents focus on the little baby after birth. Whenever the baby cries, the grandmothers and grand fathers become concerned that the baby remains hungry with breast milk. On the contrary, if the baby is formula fed, the grandparents usually feel more comfortable because they are able to see how much the baby has consumed."(Nurse from Gynecology and Obstetrics Department)(Zhang, K., et al., 2015) |
|  |  | "This is the only baby I have, I would definitely breastfeed him. I learned how to continue breastfeeding online after returning to work, such as squeezing milk, storing milk."(Li, J.P., et al., 2014) |
|  |  | "I know the HongKong government really wants to promote breastfeeding... But basically, there is no one to support me no matter how the government promotes it to us… Something more should be done. It would be very good to have a nurse coming to our homes although I know it's difficult to do that due to the shortage of nurses. But I think it's a good idea." (Tarrant, M., et al., 2014) |
|  |  | "I felt that it was the responsibility of society and government. The saying 'we should build more lactation rooms' has been said repeatedly for so long time, why I can’t see a lactation room." (Zhao, Y., et al., 2018) |
|  | Implementation in workplaces |  |
|  |  | "There is one hour in total for breastfeeding breaks, half an hour in the morning and half an hour in the afternoon. ... It is enough for me. … I drive home to breastfeed my baby every day."(Chen, J.W., et al., 2019) |
|  |  | "Maternity leave and breastfeeding leave are permitted in my work unit. As for the breastfeeding leave, mothers needn’t work on the night shift and there is one hour per day for breastfeeding within one year postpartum." "When the maternity leave is off, there is half an hour at noon per day for breastfeeding so I can return home to breastfeed my child."（Yan, N., et al.，2018） |
|  |  | "My work unit positively implements the policy of breastfeeding leave. Women will not be asked to go on business trips and will be given preferential treatment on the term of work stuff during the lactation period."（Yan, N., et al.，2018） |
|  |  | "Being very busy in the day shift, I was not able to find time to express milk. This feeling was uncomfortable. However,I tried my best to find time to express milk, in order to collect more milk for my baby." "Then at seven o'clock I would express milk, and I would express my breasts from some time past twelve to around two o'clock in the early morning for about 300-400ml before going to sleep Thus, I could extend more time for my sleep." "When I went back home, my baby was still sleeping, I expressed all of the milk out of my breasts. I felt tired.My family didn't interupt my sleep and sometimes I couldn't breastfeed my baby well on the graveyard shift." "It was more tiring when working on graveyard shifts. If I was lax in expressing my milk. But when the volume of breast milk in the refrigerator was low, I would work very hard to express again." (Nurses on rotational shifts)(Wu, C.H., et al., 2008) |
|  |  | "Because I have night shifts from time to time, if I return to work, it means that I have to wean." "The inconvenience at workplace was one of the reasons. I worked at the first floor of the hall so I had to help people with lots of questions even though I had my own office room. It would be very inconvenient for me to suspend work from time to time and go inside to breastfeed him. I think it would be better for me to only breastfeed him at home."(Chen, J.W., et al., 2019) |
|  |  | "I would go to milk my breasts once CPR was finished. If I was busy at work, I could not leave whenever I felt the need" (Nurses on rotational shifts)(Wu, C.H., et al., 2008) |
|  |  | One mother was an accountant in a private car company where expressing during work hours was not supported. Finally, she thought it was too hard to express at work. By the end of the fourth postnatal month, she only breastfed at night because she thought ‘breastfeeding is convenient at night’.(Chang, S.-m., et al., 2013) |
|  |  | "It is challenging to find time to express milk whilst working, also not easy to find a place to express milk." (Zhang, K., et al., 2015) |
|  |  | "Is it not feeding my child before and after work? I can't feed (breastfeed) at other times. So some time before the end of maternity leave, I started to give my child formula gradually in advance, fearing that the baby will not adapt to a sudden change to formula milk powder."(Li, J.P., et al., 2014) |
|  |  | "The labor union invited specialized persons to deliver lectures about breastfeeding and childrearing, from which I can acquire a lot." "My work unit invited specialists for lectures of women healthcare on the Women’s Day, which was concerned with knowledge of breastfeeding, I learned benefits of breastfeeding"（Yan, N., et al.，2018） |
|  |  | "I need to bring the pump, ice bag, and breast milk bag to the company. It feels like taking small luggage to work every day, especially I have to take metro, it is really a hassle for me" (Zhang, Y., et al., 2018) |
