# Supplementary material for: Factors influencing breastfeeding practices in China: A meta‐aggregation of qualitative studies
Source: Matern Child Nutr. 2021 Aug 6;17(4):e13251. doi: 10.1111/mcn.13251 (PMC8476444; doi:10.1111/mcn.13251)
Supplement: Supplementary file 5 — Table S4 CERQual Qualitative Evidence Profile [file MCN-17-e13251-s001.docx]

Supplementary table 4 CERQual Qualitative Evidence Profile

| Summary of the review finding | Studies contributing to the review finding | Methodological limitations | Coherence | Adequacy | Relevance | CERQual assessment of confidence in the evidence | Explanation of CERQual assessment |
| --- | --- | --- | --- | --- | --- | --- | --- |
| 1. Perceptions: Mothers commonly perceive that breast milk is equally or even less nutritious than formula, or breast milk supply is insufficient. Many women recognized that breastfeeding is beneficial for infant and maternal health. Some women found breastfeeding more difficult than expected. | Chen et al., 2016; Gao et al., 2016; Hanser & Li, 2017; Jiang et al., 2012; Li et al., 2014; Ouyang et al., 2016; Tarrant et al., 2014; Wu et al., 2017; Yan et al., 2018; H. Yang et al., 2016; Yu et al., 2018; Zhang et al., 2015; Zhang et al., 2018 | Moderate methodological limitations( 5 studies with minor, 6 studies with moderate methodological limitations (unclear recruitment strategy, data collection methods) and 1 study with serious methodological limitations (insufficiently rigorous data analysis)) | No or very minor concerns about coherence | No or very minor concerns about data adequacy (The data that this finding was based on were often relatively superficial. However, the finding was a relatively simple, primarily descriptive finding.) | Minor concerns about relevance (Geographical spread: 2 studies in northern cities, 11 studies in southern cities. One study with indirect partial reported paternal views of breastfeeding.) | High confidence | Moderate methodological limitations and minor concerns regarding relevance. No or very minor concerns about coherence and adequacy. |
| 2. Self-efficacy: Women’s breastfeeding practice was influenced by their own and others' experiences with breastfeeding, physical factors, challenges encountered when returning to work. | Chen et al., 2019; Chen et al., 2016; Gao et al., 2016; Li et al., 2014; Tarrant et al., 2014; Wu et al., 2017; Yang et al., 2015; Yu et al., 2018; Zhang et al., 2015; Zhang et al., 2018 | Moderate methodological limitations( 3 studies with minor, 6 studies with moderate methodological limitations (unclear recruitment strategy, data collection methods)) | No or very minor concerns about coherence | No or very minor concerns about data adequacy (The data that this finding was based on were often relatively superficial. However, the finding was a relatively simple, primarily descriptive finding.) | Minor concerns about relevance (Geographical spread: 2 studies in northern cities, 8 studies in southern cities. One study with indirect partial reported paternal views of breastfeeding.) | High confidence | Moderate methodological limitations and minor concerns regarding relevance. No or very minor concerns about coherence and adequacy. |
| 3. Social expectation: Most mothers reported the positive attitudes of co-workers to breastfeeding. In some cases, attitudes of employers were not supportive. Some mothers mentioned unfriendly atmosphere about breastfeeding in public. | Chang et al., 2014; Chen et al., 2019; Li et al., 2014; Wu et al., 2008; Yan et al., 2018; Zhang et al., 2015; Zhang et al., 2018 | Moderate methodological limitations( 3 studies with minor and 3 studies with moderate methodological limitations (unclear recruitment strategy, data collection methods)) | No or very minor concerns about data adequacy | Minor concerns about adequacy (3 studies offered thin data) | Minor concerns about relevance (Geographical spread: 1 study in a northern city, 5 studies in southern cities.) | Moderate confidence | Moderate methodological limitations and minor concerns regarding relevance and adequacy. No or very minor concerns about coherence. |
| 4. Social support: Almost all mothers described that they had received physical or psychological support to breastfeeding from their spouse. Both encouragement and discouragement of breastfeeding from mother and mother-in-law were common, mostly related to their own experiences and perceptions of feeding. | Chang et al., 2014; Chen et al., 2019; Chen et al., 2016; Gao et al., 2016; Ho & McGrath, 2011; Li et al., 2014; Tarrant et al., 2014; H. Yang et al., 2016; Yu et al., 2018; Zhang et al., 2015; Zhang et al., 2018 | Moderate methodological limitations( 3 studies with minor and 3 studies with moderate methodological limitations (unclear recruitment strategy, data collection methods)) | No or very minor concerns about data coherence | No or very minor concerns about data adequacy | Minor concerns about relevance (Geographical spread: 2 studies in northern cities, 9 studies in southern cities.) | High confidence | Moderate methodological limitations and minor concerns regarding relevance. No or very minor concerns about coherence and adequacy. |
| 5. General medical and health services: One nurse and many mothers reflected a lack of effective guidance on breastfeeding skills when postpartum women were in the hospital after delivery. Many participants reported the positive role of outpatient breastfeeding consultations, especially on breastfeeding confidence. In contrast, some women reported that medical staff in the community level lacked professional guidance on breastfeeding. | Chen et al., 2016; Gao et al., 2016; Jiang et al., 2012; Li et al., 2014; Ouyang et al., 2016; Tarrant et al., 2014; Wu et al., 2017; Yang et al., 2011; Yang et al., 2015; Yu et al., 2018; Yu et al., 2013; Zhang et al., 2015; Zhang et al., 2018 | Moderate methodological limitations( 5 studies with minor, 6 studies with moderate methodological limitations (unclear recruitment strategy, data collection methods) and 1 studies with serious methodological limitations ( insufficiently rigorous data analysis)) | No or very minor concerns about coherence | No or very minor concerns about data adequacy. | Minor concerns about relevance (Geographical spread: 2 studies in northern cities, 12 studies in southern cities.) | High confidence | Moderate methodological limitations and minor concerns regarding relevance. No or very minor concerns about coherence and adequacy. |
| 6. Services with Chinese characteristics: Maternity matron and cuirushi were considered helpful to breastfeeding, while in some cases they were reported to let mothers feed the baby infant formula, or delivered misinformation of breastfeeding. | Wu et al., 2017; Yu et al., 2018; Zhang et al., 2018; Chang et al., 2014; Zhang et al., 2015; Ouyang et al., 2016; | Moderate methodological limitations( 2 studies with minor, 2 studies with moderate methodological limitations (unclear recruitment strategy, data collection methods) and 1 studies with serious methodological limitations ( insufficiently rigorous data analysis)) | No or very minor concerns about coherence | Moderate concerns about adequacy (3 studies offered thin data) | Moderate concerns about relevance ( partial relevance as 1 study from a first-tier city, 4 studies from second-tier cities and 1 study from a third-tier city) | Low confidence | Moderate methodological limitations, and moderate concerns regarding adequacy and relevance. No or very minor concerns about coherence. |
| 7. Facilities: The lack of designated public places for breastfeeding, unavailable places for expressing breastmilk and refrigerators for storing the milk in workplaces was listed as barriers to breastfeeding. | Chang et al., 2014; Chen et al., 2019; Hu et al., 2013; Jiang et al., 2012; Li et al., 2014; Wu et al., 2008; Yan et al., 2018; Zhang et al., 2015; Zhang et al., 2018; Zhao et al., 2018 | Moderate methodological limitations( 5 studies with minor, 3 studies with moderate methodological limitations (unclear recruitment strategy, data collection methods) and 1 studies with serious methodological limitations ( inappropriate study design)) | No or very minor concerns about coherence | No or very minor concerns about data adequacy. | Minor concerns about relevance (Geographical spread: 1 study in a northern city, 8 studies in southern cities.) | Moderate confidence | Moderate methodological limitations and minor concerns regarding relevance. No or very minor concerns about coherence and adequacy. |
| 8. Government’s enactment of policies: Although mothers referred to maternity leave and breastfeeding leave policies as promoting breastfeeding, many mentioned that a one-hour breastfeeding break was not long enough. An obstetrics nurse and a mother both mentioned that the one-child policy and the increased involvement of the extended family in feeding a single child act as a barrier to breastfeeding. | Zhao et al., 2018; Tarrant et al., 2014; Hanser & Li, 2017; Hu et al., 2013; Li et al., 2014; Zhang et al. 2015; Chen et al., 2019 | Moderate methodological limitations( 3 studies with minor, 3 studies with moderate methodological limitations (unclear recruitment strategy, data collection methods) and 1 study with serious methodological limitations (inappropriate research design) | Minor concerns about coherence (some concerns about the fit between the data from primary studies and the review finding) | Minor concerns about adequacy (7 studies that together offered moderately rich data) | Moderate methodological limitations( 3 studies with minor, 3 studies with moderate methodological limitations (unclear recruitment strategy, data collection methods) and 1 study with serious methodological limitations (inappropriate research design) | Moderate confidence | Moderate methodological limitations, moderate concerns regarding relevance, and minor concerns about adequacy and coherence. |
| 9. Implementation of policies in the workplace: Many mothers reported breastfeeding breaks could not be guaranteed mainly due to difficulties in finding places and time to breastfeed during busy work schedules. | Chang et al., 2014; Chen et al., 2019; Li et al., 2014; Wu et al., 2008; Yan et al., 2018; Zhang et al., 2015; Zhang et al., 2018 | Moderate methodological limitations( 3 studies with minor, 3 studies with moderate methodological limitations (unclear recruitment strategy, data collection methods) | No or very minor concerns about coherence | No or very minor concerns about adequacy | Minor concerns about relevance (Geographical spread: 1 study in a northern city, 5 studies in southern cities.) | Moderate confidence | Moderate methodological limitations, minor concerns regarding relevance. No or very minor concerns about adequacy and coherence. |
